# Endoglin and MMP14 Contribute to Ewing Sarcoma Spreading by Modulation of Cell–Matrix Interactions

**DOI:** 10.3390/ijms23158657

**Published:** 2022-08-04

**Authors:** Pilar Puerto-Camacho, Juan Díaz-Martín, Joaquín Olmedo-Pelayo, Alfonso Bolado-Carrancio, Carmen Salguero-Aranda, Carmen Jordán-Pérez, Marina Esteban-Medina, Inmaculada Álamo-Álvarez, Daniel Delgado-Bellido, Laura Lobo-Selma, Joaquín Dopazo, Ana Sastre, Javier Alonso, Thomas G. P. Grünewald, Carmelo Bernabeu, Adam Byron, Valerie G. Brunton, Ana Teresa Amaral, Enrique De Álava

**Affiliations:** 1 Institute of Biomedicine of Sevilla (IBiS), Virgen del Rocio University Hospital/CSIC/University of Sevilla/CIBERONC, Molecular Pathology of Sarcomas, 41013 Seville, Spain; 2 Department of Normal and Pathological Cytology and Histology, School of Medicine, University of Seville, 41009 Seville, Spain; 3 Cancer Research UK Edinburgh Centre, Institute of Genetics and Cancer, University of Edinburgh, Edinburgh EH4 2XU, UK; 4 Clinical Bioinformatics Area, Fundación Progreso y Salud (FPS), CDCA, Hospital Virgen del Rocío, 41013 Seville, Spain; 5 Unidad Hemato-oncología Pediátrica, Hospital Infantil Universitario La Paz, 28046 Madrid, Spain; 6 Unidad de Tumores Sólidos Infantiles, Instituto de Investigación de Enfermedades Raras, Instituto de Salud Carlos III (IIER-ISCIII), 28029 Madrid, Spain; 7 Centro de Investigación Biomédica en Red de Enfermedades Raras, Instituto de Salud Carlos III (CB06/07/1009; CIBERER-ISCIII), 28029 Madrid, Spain; 8 Division of Translational Pediatric Sarcoma Research, German Cancer Research Center (DKFZ), German Cancer Consortium (DKTK), 69120 Heidelberg, Germany; 9 Hopp-Children’s Cancer Center Heidelberg (KiTZ), 69120 Heidelberg, Germany; 10 Institute of Pathology, Heidelberg University Hospital, 69120 Heidelberg, Germany; 11 Centro de Investigaciones Biológicas Margarita Salas, Consejo Superior de Investigaciones Científicas (CSIC), 28040 Madrid, Spain; 12 Division of Molecular and Cellular Function, School of Biological Sciences, Faculty of Biology, Medicine and Health, University of Manchester, Manchester M13 9PT, UK

**Keywords:** Ewing sarcoma, endoglin, mechano-transduction, extracellular matrix

## Abstract

Endoglin (ENG) is a mesenchymal stem cell (MSC) marker typically expressed by active endothelium. This transmembrane glycoprotein is shed by matrix metalloproteinase 14 (MMP14). Our previous work demonstrated potent preclinical activity of first-in-class anti-ENG antibody-drug conjugates as a nascent strategy to eradicate Ewing sarcoma (ES), a devastating rare bone/soft tissue cancer with a putative MSC origin. We also defined a correlation between ENG and MMP14 expression in ES. Herein, we show that ENG expression is significantly associated with a dismal prognosis in a large cohort of ES patients. Moreover, both ENG/MMP14 are frequently expressed in primary ES tumors and metastasis. To deepen in their functional relevance in ES, we conducted transcriptomic and proteomic profiling of in vitro ES models that unveiled a key role of ENG and MMP14 in cell mechano-transduction. Migration and adhesion assays confirmed that loss of ENG disrupts actin filament assembly and filopodia formation, with a concomitant effect on cell spreading. Furthermore, we observed that ENG regulates cell–matrix interaction through activation of focal adhesion signaling and protein kinase C expression. In turn, loss of MMP14 contributed to a more adhesive phenotype of ES cells by modulating the transcriptional extracellular matrix dynamics. Overall, these results suggest that ENG and MMP14 exert a significant role in mediating correct spreading machinery of ES cells, impacting the aggressiveness of the disease.

## 1. Introduction

Metastasis is a highly complex process with several coordinated and balanced steps and remains the main cause of mortality in cancer patients [1,2]. Multiple proteins have been identified as active players in the metastatic process. Herein, we focus on endoglin (ENG, CD105) and matrix metalloproteinase 14 (MMP14). ENG is a MSC marker reported to be highly expressed by active endothelial cells during angiogenesis [3,4,5] as well as in an extensive number of cancers and is associated with poor prognosis [6,7,8,9,10]. This transmembrane glycoprotein is a co-receptor of the transforming growth factor (TGF) β superfamily [11]. Endothelial ENG favors proliferation by promoting downstream cascade activation via SMAD1/5 while indirectly impairing ALK5/TGFβRII-regulated SMAD2/3 phosphorylation [12]. Additionally, ENG plays an important role in endothelial cell plasticity and migration by remodeling actin filaments [13,14]. Hence, ENG participates in the formation of actin stress fibers (ASF), i.e., macromolecules composed of 10–30 actin filaments, directly implicated in cellular mechano-transduction [15]. ASF typically bind to focal adhesion sites (FA), the multi-protein scaffolds responsible for connecting the extracellular matrix (ECM) with intracellular signaling pathways [16]. In fact, ENG is tightly associated with some FA components to organize actin filaments and regulate migration in endothelial cells [13,17]. ECM substrates firstly interact with integrins (ITGs) located at FA on the cell membrane. Substrates such as fibronectin (FN) and gelatin (GL) are ITG-dependent, whereas poly-D-Lysine (PDL) is a non-ITG-binding-dependent substrate.

We recently reported that anti-ENG drug conjugates are a promising option for the treatment of Ewing sarcoma (ES) [18]. ES is a highly aggressive bone/soft tissue neoplasm with a putative mesenchymal stem cell (MSC) origin, driven by a chromosomal translocation between EWSR1 and an ETS gene (FLI1 in about 85% cases), which is considered the hallmark of the disease [19]. Metastasis remains the most unfavorable prognostic factor in ES. As previously shown by our group, ES patients exhibit a predominantly positive ENG/MMP14 co-expression [18]. The link between ENG and MMP14 was shown to be relevant in the shedding of ENG from the cell membrane [20]. Moreover, MMP14 is a key factor promoting activation of other MMPs and participating in tissue remodeling, in favor of a pro-metastatic phenotype [21,22]. We deepened the study of ENG and MMP14 in ES patient samples and engineered in vitro and in vivo mouse models. We engineered overexpressing and underexpressing models of ENG in vitro and in vivo, which were studied by transcriptomics and proteomics and functionally validated. Two knockout models of MMP14 were also generated and analyzed by transcriptomics. Finally, we defined potential ENG intracellular interactors. Altogether, we show that both ENG and MMP14 play a relevant role in ES cell–matrix interaction, promoting ES dissemination.

## 2. Results

### 2.1. ENG and MMP14 Expression Predicts Prognosis of ES Patients

**Figure 1:**
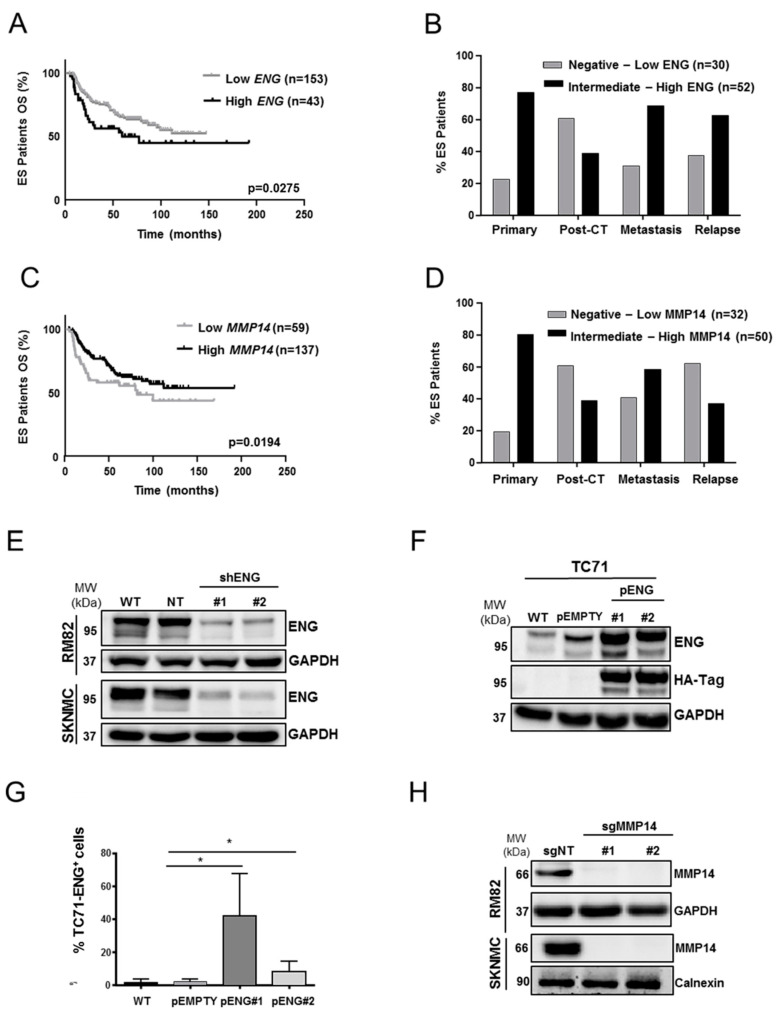
ENG and MMP14 are associated with poor prognosis in ES. Development of ES experimental models silencing/overexpressing ENG and lacking MMP14. (**A**) Kaplan–Meier curve associated with the survival of ES primary patients with high and low ENG expression (n = 196; *p* = 0.0275). (**B**) Percentage of ES patients presenting a negative-low or intermediate-high score of *ENG* expression in the local cohort of patients stratified according to clinical stage (n = 83). (**C**) Kaplan–Meier curve associated with the survival of ES primary patients with high and low *MMP14* expression (n = 196; *p* = 0.0194). (**D**) Percentage of ES patients presenting a negative-low or intermediate-high score for MMP14 expression in the local cohort of patients stratified according to the clinical stage (n = 83). Grehan–Breslow–Wilcoxon test was used to analyze patient survival. (**E**) Generation of knockdown models of ENG in the RM82 and SK-N-MC cell lines and candidate clones were confirmed by WB. (**F**) Generation of TC71 ENG-overexpressing models, as confirmed by WB for ENG and HA-tag. (**G**) Validation of ENG overexpression at the cell membrane in the TC71 model. (**H**) Validation by WB of the MMP14 knockout in two clones derived from the SK-N-MC and RM82 cell lines. Paired two-tailed Student’s *t*-test was used to evaluate statistical significance (* *p* < 0.05).

We previously reported that ENG and MMP14 are variably expressed in ES patients (n = 43) [23]. The first aim of this work was to perform a deeper evaluation of ENG and MMP14 expression in a larger cohort of ES patients. Initially, we assessed the overall survival (OS) in a large series of patients from public datasets (n = 196). A significantly different overall survival was observed when high and low mRNA levels of *ENG* were stratified according to the 78th percentile cut-off (*p* = 0.0275) (Figure 1A and Figure S1A), which was the best stratification percentile. According to our previous work, a positive correlation was found between *ENG* and *MMP14* mRNA levels (Figure S1B) ENG protein expression in our local cohort (n = 82) was analyzed by IHC. Samples were stratified according to: primary tumor (n = 35; 42%), post chemotherapy (post-CT) (n = 23; 28%), metastasis (n = 16; 20%), and relapse (n = 8; 10%). Tumor samples presented extensive homogenous protein levels of both ENG/MMP14 on the TMA sections and thus were evaluated by intensity only (Table S1A,B). When patients were grouped into negative-low or intermediate-high, a larger percentage of patients presented intermediate-high ENG expression in the primary tumor, metastasis, and relapse (Figure 1B; Table S1C). However, no differences were observed in OS of ES patients (local cohort) regarding ENG expression in primary tumors. It is important to mention that follow-up was only available for 35 patients. Strikingly, low mRNA levels of *MMP14* from public data were associated with a worse outcome in ES patients (*p* = 0.0194) (Figure 1C) when the 30th percentile was considered as the best stratification cut-off. At the protein level, intermediate-high MMP14 expression was higher in primary (80%) and metastatic (63%) samples (Figure 1D; Table S2A–C). Again, no correlation was found in terms of OS in this local cohort of 35 patients with follow-up. These data suggest that ENG might be promoting malignancy and that further functional assays are needed to evaluate the roles of both ENG and MMP14 in ES. 

### 2.2. ENG Affects the Clonogenic Capacity and Neo-Angiogenesis of ES Cells

To explore the role of ENG in ES cells, ES cell lines with high (RM82 and SK-N-MC) and low (TC71) ENG expression were selected [18]. Both RM82 and SK-N-MC cells were used to generate ENG-knockdown models. The TC71 cell line was used to develop an upregulated model of transmembrane ENG by plasmid integration (pENG). (Figure S2) ENG downregulation was confirmed by Western blotting (WB) and qRT-PCR (Figure 1E and Figure S1C,D, respectively), whereas reduction of transmembrane ENG was observed in >40% of cells, as confirmed by FACS on the candidate clones (Figure S1E,F). ENG overexpression in the TC71 cell line was confirmed by WB and FACS (Figure 1F–G). Given the relevance of transmembrane ENG in cell–cell and cell–matrix interactions, only candidate clones where ENG was clearly over/downregulated, as detected by FACS, were eventually selected for transcriptomic, proteomic, and functional studies. Overexpression of transmembrane ENG was higher in one clone pENG#1 (around 50% of cells overexpressing ENG) when compared to clone pENG#2 (around 20% of cells overexpressed ENG) (Figure 1G). Additionally, MMP14-knockout models were generated in the RM82 and SK-NMC cell lines. MMP14 gene editing was confirmed by Sanger sequencing, and loss of protein was detected by WB (Figure 1H).

**Figure 2:**
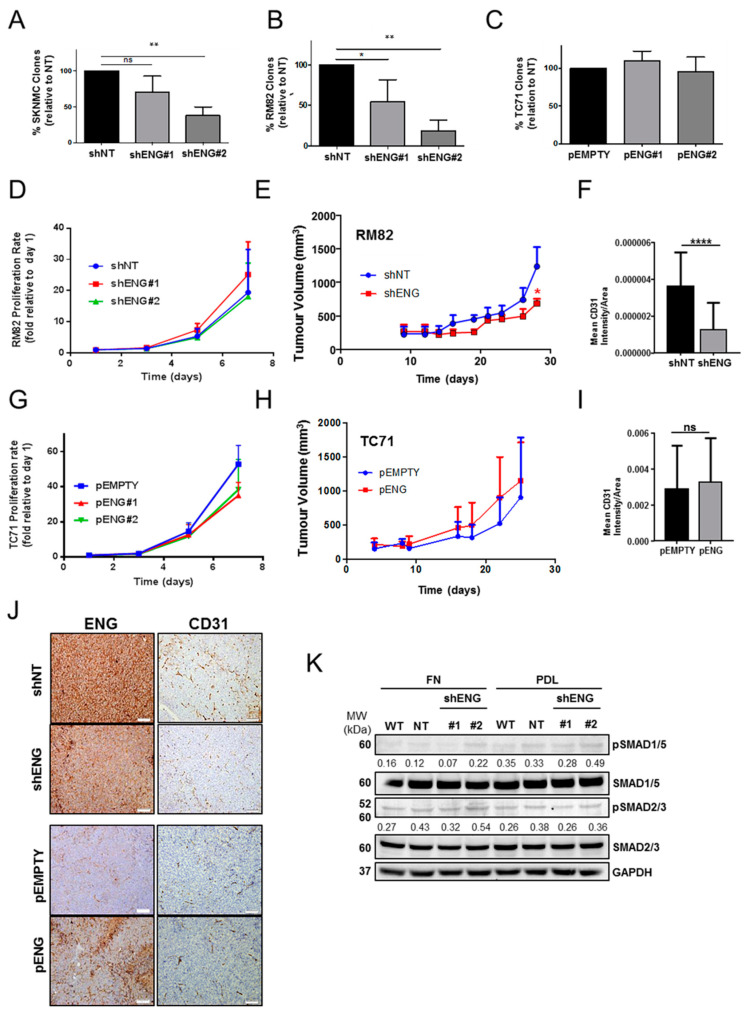
ENG downregulation in ES cells impairs clonogenicity, without affecting cell proliferation, independently of SMAD phosphorylation. (**A**–**C**) The clonogenic capacity was impaired when ENG was downregulated in both RM82 (**A**) and SK-N-MC (**B**) cells, while there was no improved clonogenic potential in the upregulated model (**C**). (**D**) In vitro proliferation was not affected by the downregulation of ENG in the RM82 model. (**E**) In vivo, the downregulation of ENG slightly impaired the ES tumor growth, reaching significance at the endpoint. (**F**) Expression of the vessel marker CD31 was significantly decreased when ENG was downregulated in the shENG model in vivo, as determined by IHC and quantified by Fiji. (**G**,**H**) In vitro cell proliferation (**G**) and in vivo tumor growth (**H**) were not affected by ENG upregulation in the TC71 pENG model. (**I**) Differences in CD31 expression were not significant in the ENG-overexpressing model in vivo, as detected by IHC and quantified by Fiji. (**J**) Tumoral downregulation/upregulation of ENG were maintained in the shENG and the pENG models in vivo, respectively, as determined by IHC at the end of study. CD31 staining was lower when ENG was downregulated but unaffected in the overexpressing condition. (**K**) No changes on SMAD2/3 phosphorylation were observed in the presence of GL/FN or in the absence of ENG, as determined by WB quantification. Statistical significance was evaluated by paired two-tailed Student’s *t*-test (ns, not significant; * *p* < 0.05; ** *p* < 0.01; and **** *p* < 0.0001).

**Figure 3:**
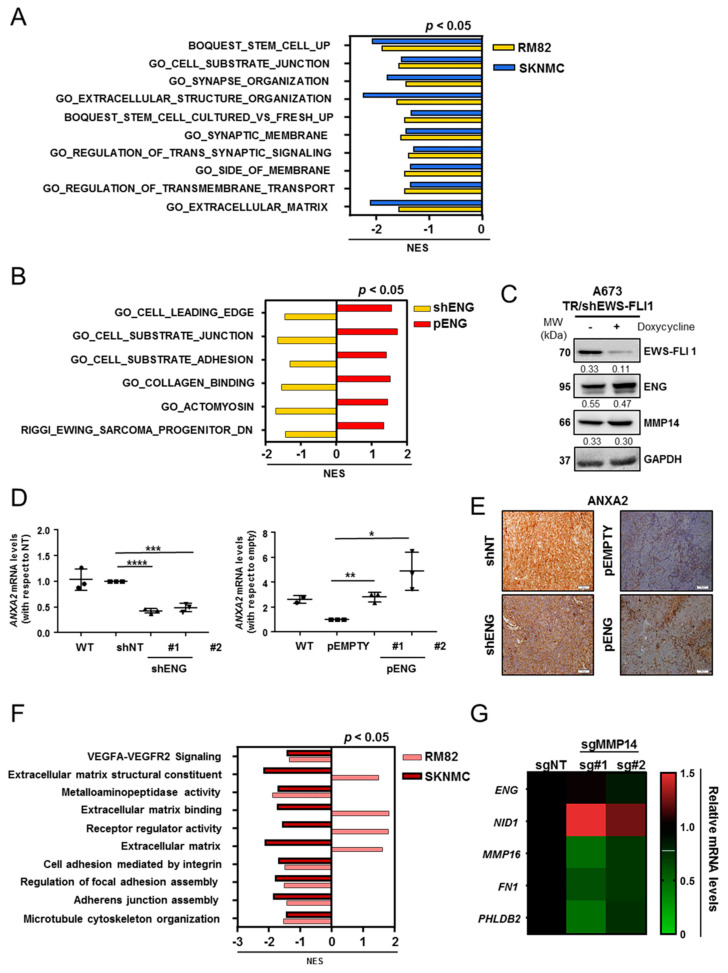
ENG and MMP14 regulate transcriptional pathways related to cell migration and cell–substrate adhesion in ES cells. (**A**) Joint transcriptomic analysis of the shENG models from RM82 and SK-N-MC revealed common downregulated GO terms related to cell–substrate adhesion and ECM organization. (**B**) Joint transcriptomic analysis of RM82-ENG-downregulated model and TC71-ENG-upregulated model showed significantly inverse gene signatures regarding cell–substrate junction and adhesion and ECM organization. (**C**) Expression of ENG and MMP14 was not dependent on EWS-FLI1 expression, as detected by WB analysis. (**D**) qRT-PCR validation of ANXA2 dysregulation on the RM82 shENG model (#1 and #2 clones) versus the shNT control and on the TC71 pENG model (#1 and #2 clones) versus the pEMPTY control. (**E**) The downregulated and overexpressed levels of ANXA2 were validated in vivo in the shENG and pENG models, respectively. (**F**) Common gene categories were observed when transcriptomic data from RM82 sgMMP14 and SK-N-MC sgMMP14 were evaluated by GSEA. The most significant common pathways included ECM structural constituent, metalloaminopeptidase activity, and ECM binding. (**G**) qRT-PCR validation of the transcriptomic analysis of RM82 sgMMP14 confirmed the gene deregulation of the sgMMP14 model. FN1 and PHLDB2 were significantly downregulated in both RM82 shMM14 clones. Statistical significance was evaluated by paired two-tailed Student’s *t*-test in (**D**) (ns, non-significant; * *p* < 0.05; ** *p* < 0.01; *** *p* < 0.001; and **** *p* < 0.0001). NES, Normalized Enrichment Score.

Upon validation of the in vitro models, we studied the effects on clonogenicity. The clonogenic capacity was impaired in both RM82 and SK-N-MC cell lines upon shENG knockdown after 10/14 days growing at low confluence (Figure 2A,B and Figure S3A,B). However, no ENG-dependent impact was observed in the TC71 pENG model (Figure 2C and Figure S3C). These results suggest that ENG is involved in processes such as self-renewal and cell–cell and/or cell–substrate adhesion in cells with high basal ENG levels. Interestingly, in vitro cell–cell attachment was not affected (Figure S3D). As expected, no significant differences were observed in terms of proliferation or cell cycle in the shENG models (Figure 3D and Figure S3E,F). RM82 shENG spheroids showed a slight increase proliferation rate in the absence of ENG (Figure S2G). We developed a double flank nude mouse xenograft model using shNT/shENG ES cells to evaluate the role of ENG in vivo (Figure 2E). No significant ENG-dependent differences were found in the tumoral outgrowth; nonetheless, tumor volume on the shENG arm was lower at the endpoint of the assay. At the end of the study, ENG was quantified by IHC, and downregulation was confirmed (Figure 2J and Figure S3H). Since endothelial ENG is crucial in neoangiogenesis, the microvascular density of the tumors was evaluated by IHC staining of murine CD31, a specific endothelial cell marker. We observed a significant reduction in CD31 staining in the shENG tumors (Figure 2F,J, upper panel). Likewise, no differences on proliferation were observed in the pENG model in vitro (Figure 2G) or in vivo (Figure 2H) with respect to the control group. ENG overexpression was also quantified at the end of the study by IHC (Figure 2J, bottom panel and Figure S3I). No significant differences in terms of CD31 expression were found in the pENG model (Figure 2I,J, lower panel). This suggests that ENG may be directly or indirectly interacting with the tumor microenvironment by increasing microvascular density, thus favoring tumor growth. Moreover, in cells without basal ENG expression, this dependency is irrelevant for neoangiogenesis.

### 2.3. Comprehensive Transcriptomic Studies Suggest That ENG Is Associated with ES Cell Migration and Substrate Adhesion

ENG is a co-receptor of the TGFβ superfamily that regulates TGFβ signaling by interacting with TGFβRII, a well-known suppressed target by the ES fusion protein [23]. To further confirm that the TGFβ pathway is downregulated, we evaluated the phosphorylation of SMAD1/5 and SMAD2/3 as downstream activators of TGFβRII cascade. As expected, no differences were found when ENG-knockdown cells were seeded on FN, an ITG-dependent substrate, or PDL, a non-dependent ITG-binding substrate. (Figure 2K). Moreover, we screened the mRNA expression of other members of the TGFβ signaling pathway potentially associated with ENG in a set of ES cell lines; however, we observed that their expression was mostly homogeneous and low (Figure S4). These results confirm that the role of ENG in ES is independent from the TGFβ pathway. To evaluate the ENG-dependent transcriptional effects, we performed transcriptomic analyzes of the downregulated models, RM82 (#GSE173145) and SK-N-MC (#GSE173148), and the upregulated model, TC71-pENG (#GSE173153), using Gene Set Enrichment Analysis (GSEA). Common significantly downregulated gene ontologies (GO) terms were found in both RM82 and SK-N-MC ENG-knockdown models, mostly associated with ECM remodeling and cell–substrate junctions (Figure 3A; Table S3). Additionally, the RM82-shENG and the TC71-pENG transcriptomic profiles were cross-analyzed to further validate these findings. An inverse Normalized Enrichment Score (NES) was found amongst common gene categories and was related to cell-shape plasticity and substrate adhesion (Figure 3B; Table S4). A common inversely deregulated gene signature was generated between the RM82 shENG and TC71 pENG profiles (Figure S5A) and the candidate genes belonging to these categories: MMP14, the basement membrane protein nidogen 1 (NID1), the calcium-dependent phospholipid-binding protein annexin 2 (ANXA2), and the cytoskeleton-associated protein palladin (PALLD) (Figure S5B,C).

Recent evidence shows that ES dissemination is mediated by the direct modulation of the fusion protein expression, the driver of ES. Thus, a transcriptionally high EWS-FLI1 status would facilitate a steadier and more proliferative cell program, whereas a low-EWS-FLI1 status would promote invasiveness [24,25]. To define if ENG and/or MMP14 are associated with the EWS-FLI1 signature, we evaluated the expression of ENG in public RNAseq data for ES cells expressing high or low EWS-FLI1 [26]. We observed that both ENG and MMP14 transcripts were upregulated when EWS-FLI1 was decreased (Figure S6A). To further confirm the findings at the protein level, we used the A673/TR/shEWS-FLI model with inducible downregulation of EWS-FLI1 upon doxycycline treatment. We observed no significant increase in the protein levels of either ENG or MMP14 when EWS-FLI1 was downregulated (Figure 3C, Figure S6B). These results imply that both ENG and MMP14 expression levels are not regulated by the fusion protein.

In parallel, we used the in-silico tool HiPathia to model signaling pathway deregulation from the transcriptomic data of both RM82- and SK-N-MC shENG [27] (http://ngsresults.clinbioinfosspa.es/IBIS_Endoglina/RM82_C/pathway-viewer/ and http://ngsresults.clinbioinfosspa.es/IBIS_Endoglina/SKNMC/pathway-viewer, respectively, accessed on 17 November 2021). Transcriptomic data were transformed into activity-state estimations of the physiological signaling pathways. Some of the most significantly different signaling pathways were related to cell adhesion functions (Table S5). One of the most significant genes inversely deregulated in the shENG and pENG models was ANXA2. Members of the annexin family are direct substrates of PKC and regulate actin dynamics. ANXA2, which encodes annexin A2, was validated by qRT-PCR and at the protein level by IHC in the in vivo models (Figure 3D,E). Together, our studies showed a deregulation of the transcriptional programs related to cell migration and adhesion of ES cells.

### 2.4. MMP14 Is Involved in ECM Organization and Cell–Substrate Adhesion in ES

We used two MMP14-knockout (sgMMP14) on RM82 and SK-N-MC cell lines, by CRISPR-Cas9 gene editing. MMP14-knockout models (RM82 (#GSE173143) and SK-N-MC (#GSE173147) share common GO terms related to the metastatic potential of cancer cells, which were mostly downregulated, as evaluated by GSEA (Figure 3F; Table S6). These included metalloaminopeptidase activity or regulation of focal adhesion assembly and other GO terms associated with cell motility (*p* < 0.02). The most relevant genes commonly downregulated in RM82-/SK-N-MC-sgMMP14 were selected for validation by qRT-PCR in the RM82 model (Figure 3G; Figure S7A). These results showed that loss of MMP14 significantly modulates genes associated with the ECM, namely FN1 and NID1, when compared to the non-targeting control (*p* < 0.05). ENG expression was maintained in the absence of MMP14, as determined by qRT-PCR (Figure 3G; Figure S7A).

### 2.5. ENG Contributes to the Process of ES Cell Migration and Adhesion, whereas Loss of MMP14 Enhances Cell–Substrate Adhesion

**Figure 4:**
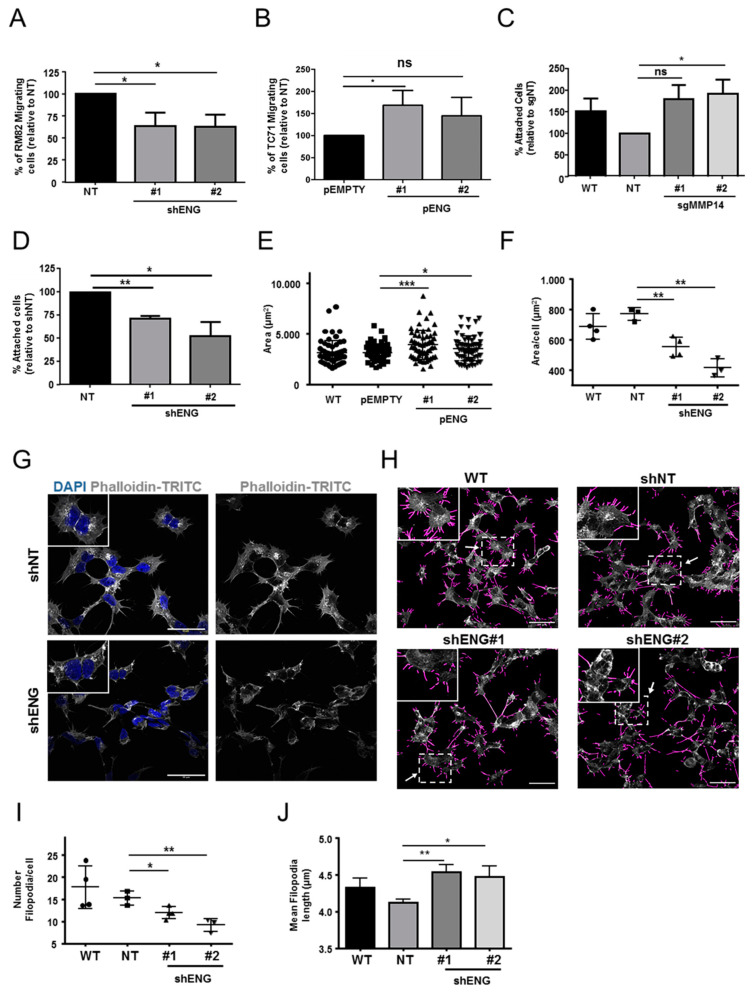
Cell migration and adhesion are positively regulated by ENG in association with actin filaments and filopodia formation in ES cells in vitro. (**A**) Cell migration assay of RM82 shENG cells in Boyden chamber. In the absence of ENG, migration was significantly reduced in comparison to the control condition. (**B**) Cell migration assay from TC71 pENG cells in Boyden chamber. Overexpression of ENG promoted migration, which was statistically significant in one of the clones. (**C**) Cell–substrate attachment assay using RM82 sgMMP14 cells on 20 µg/mL FN for 15 min. Expression of MMP14 significantly impaired attachment on FN. (**D**) Cell attachment assay from the RM82 shENG model performed on 20 µg/mL FN for 15 min. ENG downregulation was associated with lower adherence to the substrate. (**E**) Cell spreading assay was performed TC71 pENG model on 20 µg/mL FN for 1 h. Cell spreading was increased in the presence of ENG. (**F**) Quantification of filamentous actin area showed a reduced cytoplasmic location in the ENG-downregulated condition. (Grey scale bar, 50 µm.) (**G**) IF of actin filament staining by phalloidin-TRITC in RM82 shENG cells. Downregulation of ENG led to actin filament aggregates and disrupted distribution along the cytoplasm. (**H**) IF of actin filaments (gray) and identification of filopodia (purple) by Fiji (FiloQuant) in RM82 WT, shNT, and shENG cells. (**I**,**J**) The density of filopodia per cell (**I**) and the filopodia length (**J**) were measured by filopodia quantification in RM82 WT, shNT, and shENG cells. Paired (**A**,**B**,**D**) and unpaired (**C**,**E**,**F**) two-tailed Student’s *t*-test were used to determine statistical significance (* *p* < 0.05; ** *p* < 0.01; and *** *p* < 0.001).

To study if ENG is involved in the regulation of ES dissemination, cell migration assays on the RM82-shENG and TC71 pENG models were performed using Boyden chambers. RM82-shENG cells had significantly reduced migratory capacity when compared to control cells (Figure 4A). Conversely, increased migration was detected when ENG was overexpressed in the TC71-pENG model (Figure 4B). To evaluate if MMP14 directly affects ES cell adhesion, we performed a cell–substrate adhesion assay. Interestingly, an increased adhesive capacity was detected in the RM82-sgMMP14 model (*p* = 0.04 in clone #2) (Figure 4C). These results support the idea that both ENG and MMP14 might be involved in the ES metastatic process. Based on the GO terms and the in silico transcriptomic analysis, ENG-dependent cell–substrate adhesion was monitored for cells seeded on FN in a 2D approach in vitro. In terms of cell–substrate attachment, adhesion was impaired in RM82-shENG. (Figure 4D). Importantly, TC71-pENG presented higher cell spreading, as determined by mean cell area (Figure 4E, Figure S8A,B). We also determined if ENG could be present in extracellular vesicles (EVs) secreted by ES cells in vitro. EVs were isolated from the wild-type (WT) RM82 cell line by density gradient and ultracentrifugation. We evaluated the EVs enrichment by analyzing tetraspanins expression by WB (Figure S8C–E). ENG enrichment was observed in EVs isolated from the supernatants of RM82 cells growing in vitro (Figure S8C–E). These results suggest that ENG could exert a role at a distant site in the EV-regulated formation of metastasis in ES cells.

### 2.6. ENG Participates in ES Actin Remodeling

To address the results obtained from our transcriptomic studies, we studied the role of ENG in actin remodeling in ES cells. We first assessed the formation of actin filaments in the RM82-shENG and TC71-pENG models. These cells were seeded on FN and stained by phalloidin-TRITC in conditions under the same cell density, with the exception of shENG2, which presented predominantly cell aggregates (Figure S9A,B). Depleted ENG resulted in disrupted actin filament organization compared to control and higher levels of ENG (Figure 4F,G and Figures S9–S11). We next quantified filopodia by number and length. The downregulation of ENG was associated with a significantly lower number of filopodia with higher length (Figure 4H–J; Figures S10 and S11C). Overall, this suggests that ENG participates in ES cell motility, enhancing cell–substrate adhesion and promoting actin organization.

### 2.7. ENG Participates in the Activation of Signaling Pathways Related to Migration

**Figure 5:**
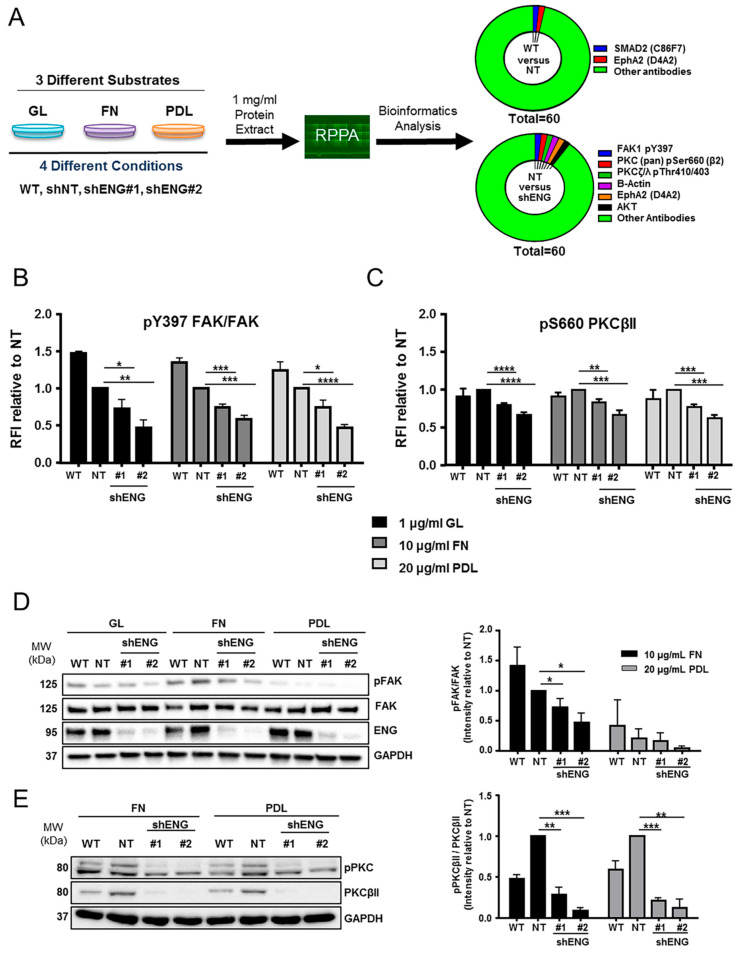
RPPA determines ENG-dependent and substrate-dependent signaling pathways in ES cells in vitro. (**A**) RM82 WT, shNT, shENG1, and shENG2 were seeded on 1 µg/mL GL, 10 µg/mL FN and 20 µg/mL PDL, and RPPA was performed after 24 h incubation at low cell density in vitro. Bioinformatics analysis depicting the most relevant deregulated proteins between WT and shNT (discarded) and between shNT and shENG conditions. (**B**) RRPA individual analysis data shows that pY397 FAK/FAK and (**C**) pS660 PKCβII are reduced in the absence of ENG independently of the substrate. RFI, relative fluorescence intensity. (**D**) In agreement with RPPA results, the identification of differential phosphorylation of Y397 residue from FAK dependent on ENG expression and seeding substrate was confirmed by WB and quantified by image J. (**E**) Likewise, the WB validation and quantification confirmed the differential phosphorylation at the S660 residue and endogenous expression of PKCβII in an ENG-dependent manner in protein extracts from shENG cells seeded on FN and PDL in vitro. Statistical significance was evaluated by paired two-tailed Student’s *t*-test (* *p* < 0.05; ** *p* < 0.01; *** *p* < 0.001; and **** *p* < 0.0001).

To better understand how ENG is involved in ES cell-shape plasticity, we performed Reverse Phase Protein Array (RPPA) analysis. We evaluated differential expression and phosphorylation status of 60 proteins associated with the process of migration (Table S7) using the RM82-shENG model seeded on three different substrates: two of them ITG-binding-dependent, namely GL and FN, and one non-ITG-binding-dependent, namely PDL. Thus, we aimed both to distinguish substrate-dependent signaling pathways and to identify potential ENG-dependent (phospho) proteins. Several significant changes were detected compared to the non-targeting condition (Figure 5A, Figures S12–S15). Among the most significant phospho-proteins, we found a decrease of both FAK and PKCβII phosphorylation when ENG was depleted independently of the substrate (Figure 5A–C). Reduction on the Y397 residue FAK phosphorylation was validated by WB (Figure 5D). In addition, substrate-dependent upregulation of Src expression was found in cells seeded on the non-ITG-dependent substrate PDL (Figure S14C). In contrast, a significant reduction in pS2448 mTOR (normalized to total mTOR) was found in cells seeded on PDL (Figure S14D). Further, phosphorylation of S660 PKCβII was reduced in the absence of ENG and associated with a downregulation of the total PKCβII levels (Figure 5E; Figure S15A–D). These results further support the role of ENG in FA signaling pathways.

### 2.8. ENG Physically Interacts with Proteins Associated with Focal Adhesion Organization in ES Cells

**Figure 6:**
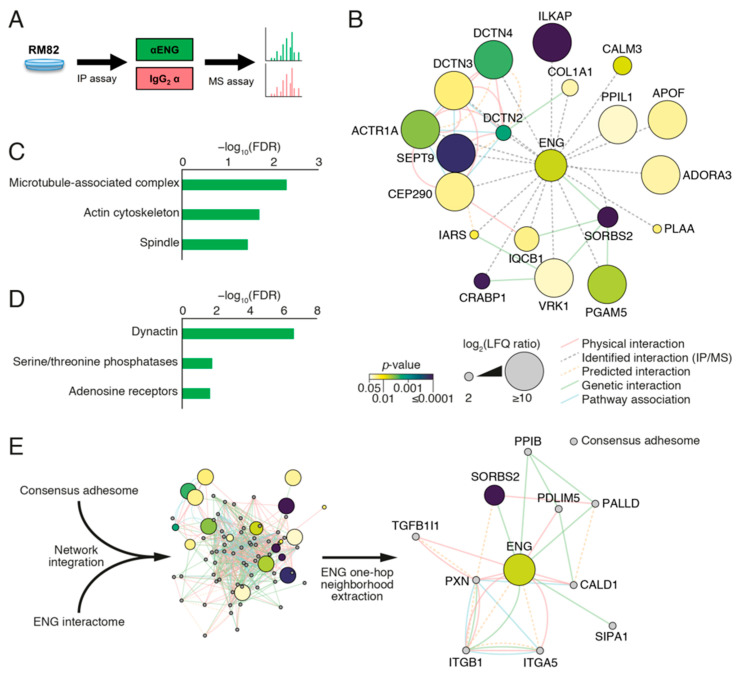
IP/MS identifies ENG-associated cytoskeletal and adhesion-related protein networks. (**A**) Workflow for the identification of potential interactors of ENG in RM82 WT cells by IP/MS. (**B**) Functional association network analysis of ENG-enriched interactors identified by IP/MS. Network edges (connecting lines) represent reported physical (red), genetic (green), pathway (blue), or predicted (dashed orange) interactions or imputed interactions detected by IP/MS (dashed gray). Nodes (circles) represent identified proteins. Node size is proportional to log_2_-transformed LFQ ratio (ENG/IgG); node fill color represents *p*-value of LFQ ratio (log_10_ scale). Nodes annotated with gene names for clarity. (**C**) GO over-representation analysis of ENG interactors identified by IP/MS, analyzing the GO cellular component domain (non-redundant database). All terms over-represented with FDR < 0.05 are shown. (**D**) Gene family enrichment analysis of ENG interactors identified by IP/MS. All terms enriched with FDR < 0.03 are shown. (**E**) In silico network integration of the published consensus adhesome with the ENG interactome identified by IP/MS. The ENG one-hop neighborhood is shown, key as for B, and gray nodes indicate consensus adhesome components (not detected by IP/MS).

To characterize the networks of proteins that physically and potentially functionally associate with ENG in ES cells, we performed an interactome analysis in the RM82 cell line using a MS/IP approach (Figure 6A). After applying a *p*-value threshold (*p*-value ≤ 0.05), peptide detection in each anti-ENG IP sample, and a fold change cut-off (ENG/IGG ≥ 2), we identified 20 potential interactors (Figure 6). Label-free quantification (LFQ) revealed ENG as one of the most enriched proteins in ENG IPs compared to IgG controls (Table S8), indicating successful isolation of ENG-associated protein complexes. To interrogate putative functional associations between proteins identified in ENG complexes, we performed interaction network analysis, incorporating reported physical, genetic, and pathway interaction data (Figure 6B; Figure S16A). The ENG interactome was enriched for cytoskeleton-associated proteins, including the actin cytoskeleton (false-discovery rate (FDR) = 0.0197) (Figure 6C). In particular, dynactin family members were highly enriched (FDR = 2.30 × 10^−7^) (Figure 6D) and formed an interconnected cluster in the ENG interaction network (Figure 6B and Figure S16A). Dynactin is a multiprotein, cytoskeleton-associated complex and a key component of the dynein motor machinery, and dynactin subunits identified in the ENG interactome included the actin-related protein α-centractin, also known as Arp1 (encoded by ACTR1A), and dynactin subunits 2 (p50; DCTN2), 3 (p24; DCTN3), and 4 (p62; DCTN4) (Table S8). Another protein highly enriched in ENG IPs was integrin-linked kinase-associated serine/threonine phosphatase 2C (ILKAP), which had one of the highest LFQ ratios compared to IgG controls and most significant *p*-values. ILKAP associates with the focal adhesion protein integrin-linked kinase (ILK). To investigate connections between ENG-associated proteins and adhesion proteins, we integrated the ENG interactome with the consensus adhesome, a database of core adhesion proteins frequently present in FAS proteomes [28,29] (Figure 6E). Interaction network analysis showed that all but two proteins (calmodulin-3 and adenosine receptor A3) in the ENG interactome were physically or functionally associated with at least one consensus adhesome protein (Figure S16B). In this integrated network, the ENG interactor Arg-binding protein 2 (ArgBP2, also known as sorbin; encoded by SORBS2) was a member of the ENG-nearest neighbor subnetwork (one-hop neighborhood), with links to adhesome proteins PALLD and paxillin, which both regulate cell adhesion to the ECM (Figure 6E). Altogether, our data suggest that ENG may physically interact with a set of proteins involved in cell adhesion, motility, and actin remodeling in ES cells.

## 3. Discussion

In our previous work, we showed the antitumor activity of ENG-targeting ADC therapies in ES [18]. Herein, we deepened our study of ENG and MMP14 in ES patients and their potential functional role in ES dissemination. The evaluation of ENG and MMP14 expression in a larger cohort of patient samples showed that high ENG levels were significantly associated with worse prognosis, suggesting that this protein could contribute tumor aggressiveness. This agrees with a previous work [9] where ENG was predominantly expressed in the endothelial compartment of the ES tumors. In rhabdomyosarcoma, ENG expression was also exclusively detected in tumor vessels [30]. Indeed, ENG is highly expressed by neovessels within the tumor in most cancer types, with very few showing expression in the tumoral compartment [31]. In our previous work, we showed that ES cells express variable levels of ENG and that ES proliferation was unaffected by ENG targeting [23]. Herein, we confirmed that ENG modulation does not affect in vitro proliferation. In vivo, however, we observed a significant reduction in tumor growth in the absence of ENG. Hence, we speculate that ENG’s interaction with the TME might promote ES growth. In fact, we found that microvessel density decreased upon ENG downregulation. We also observed impairment in clonogenicity when ENG was downregulated [32]. ENG enrichment in ES-secreted EVs further suggests ENG may play a role in distant signaling. This has been previously shown in renal tumors, where ENG present in microvesicles promoted angiogenesis and pre-metastatic niche formation [33]. Membrane-bound and soluble ENG (sENG) are involved in vascular pathophysiology mainly through the TGFβ pathway [34]. In ES, nonetheless, this pathway is not active due to the suppressed expression of TGFβRII by EWS-FLI1 [35]. TGFβ pathway activators, phospho-SMADs, were not affected by either different substrate (ITG- or non-ITG-dependent) or the presence/absence of ENG. This suggests that the role played by ENG in ES is TGFβ- independent. The invasive phenotype of ES has been attributed to EWS-FLI1 fusion protein modulation [24,25]. Here, ENG/MMP14 were not significantly affected by the EWS-FLI1 downregulation. This suggests that the role of ENG and MMP14 in migration is not dependent on the fusion protein. Moreover, both ENG and MMP14 are actively expressed by MSCs, the putative cell of origin of ES. Additionally, sENG is a direct BMP9/BMP10 antagonist, which is relevant in angiogenesis [36]. We have previously shown that ES patients express variable levels of sENG [19]. As MMP14 is the main protease responsible for ENG shedding, we engineered ES MMP14-knockout models. Transcriptionally, MMP14 absence resulted in the deregulation of processes associated with metallopeptidase activity, cell junction, and ECM organization. This is in agreement with previous work showing that MMP14 is key in the activation of MMPs such as MMP2 [21], MMP9 [22], and MMP13 [37]. Transcriptomic analysis showed that cell–substrate adhesion and migration genes were inversely affected in ENG down/upregulated models, including ANXA2, a regulator of actin dynamics [38,39]. Importantly, members of the annexin family are direct substrates of PKC [40], acting as scaffolds in membrane translocation of PKC [41,42]. Moreover, the intracellular domain of ENG presents 19 Ser/Thr residues, constitutively phosphorylated and containing at least one potential site for PKC phosphorylation [43]. Interestingly, our proteomics-based signaling analysis showed that ENG has a significant impact on PKC expression, which is known to mediate the distribution of ITG at the plasma membrane, with consequences on cell shape and motility [44]. Posterior validation showed that, in fact, ENG affects the expression of PKCβII rather than regulating the activation of the protein. PKCβ is a key protein in processes such as cell spreading and migration. PKCβ has been reported as a regulator of PDGF-stimulated vascular smooth muscle cell chemotaxis and ITGs [45]. Herein, the ENG-dependent expression of PKCβII further supports ENG involvement in the correct distribution of actin in the cytoskeleton. Functionally, we observed an ENG-dependent regulation of actin filaments and filopodia formation, further supporting our evidence that ENG participates in ES actin dynamics. ENG not only affects actin remodeling but also cell–substrate adhesion, two processes closely related. ENG has been described not only as a promoter of migration but also as an inhibitor of migration depending on the cellular context [31]. In this study, ENG promoted migration by positively regulating actin filaments in the cytoplasm and within filopodia at the cell surface. By IP/MS, we identified a subnetwork of ENG-proximal core adhesion proteins that included ITGs, paxillin, and PALLD, (Figure S17) Another highly enriched ENG interactor was ILKAP, a negative regulator of ITG signaling by phosphorylating the key focal adhesion protein ILK [46,47]. Moreover, PALLD can recruit cytoplasmic ILKAP and induce apoptosis [48]. It is important to mention that, herein, we used an anti-ENG antibody targeting the extracellular N-terminal region for IP/MS, and thus, we only focused on the intracellular interactors of ENG. As mentioned above, the RPPA-based results further support the connection between ENG and the regulation of cell dynamics at FA and cell motility in ES. Indeed, our results suggest that FAK signaling is regulated by ENG, and ENG-dependent phosphorylation of Y397 FAK reinforces the findings relative to focal adhesion, ASF, and, concurrently, cell migration.

## 4. Materials and Methods

Patient samples immunohistochemistry analysis: Public datasets from 196 primary ES tumors (GSE63157, GSE34620, GSE12102, GSE17618, and Dr. Alonso’s lab) with available clinical annotation were retrieved from *Gene Expression Omnibus* (GEO). Data were either generated on Affymetrix HGU133Plus2.0, Affymetrix HuEx-1.0-st, or Amersham/GE Healthcare CodeLink microarray chips and separately normalized by RMA using custom brainarray chip description files (CDF, v20) as previously described [49]. ComBat was assessed to adjust batch effects [27,28]. Patients were stratified by their 78th and 30th percentile according to the ENG and MMP14 expression levels, respectively. A Grehan–Breslow–Wilcoxon test was performed to calculate significance levels, considering the relatively high number of early censored events, using GraphPad PRISM (version 5) for individual genes (GraphPad Software Inc., San Diego, CA, USA). *p*-values < 0.05 were considered as statistically significant. Additionally, Tissue Micro Arrays (TMAs) from local ES patient samples (n = 82) collected at Hospital Universitario Virgen del Rocío-Institute of Biomedicine of Seville (HUVR-IBiS) Biobank were used to perform immunohistochemistry (IHC).

Cell Lines: All cell lines were grown in vitro at 37 °C and 5% CO_2_. RPMI, or EMEM for the SK-N-MC cell line, were used for cell line maintenance, supplemented with 10% FBS and 1% penicillin/streptomycin (P/S) and glutamine (all from Gibco, Thermo Fisher Scientific). RM82 and TC71 cell lines were seeded on plates pre-treated with 1 µg/mL GL from porcine skin (Sigma) to promote adherence as described by our group [19].

IHC in ES samples: Intensity (scores 0-1-2-3) and area of staining (scores 0-1-2-3) derived from ENG/MMP14 expression were evaluated by an experienced pathologist as previously described by our group (19). CD31 intensity from tumoral samples was analyzed as described elsewhere (24). Analysis of variance (ANOVA test) between groups of patients according to means of intensity was also performed. Patient survival was evaluated by Kaplan–Meier curves. Overall survival (OS) statistical analysis on the local cohort was assessed by the log-rank (Mantel–Cox) test using GraphPad Prism (version 6.0).
Flow cytometry-fluorescence-activated cell sorting-(FACS): Cells were seeded on 100-mm plates. After 24 h incubation, 80–90%-confluent cells were washed with PBS once and harvested either by trypsinization (in the case of intracellular protein analysis) or pipetting/scraping (in the case of transmembrane protein analysis). A total number of 5 × 105 cells were resuspended in 1 mL 2%-BSA PBS. Cells were centrifuged at 1500 rpm for 5 min. Cell pellets were mixed with Master Mix (anti-CD105-APC #105A-100T, Immunostep and PBS) for 30 min at room temperature, protected from light. Cells were centrifuged at 1500 rpm for 5 min and washed twice with 2%-BSA PBS. After the last wash, the suspension of cells in PBS was filtered with Falcon^®^ 70 µm Cell Strainer (BD Falcon). For cell cycle analysis, cells were incubated with 100 μg/mL Propidium Iodide and 100 μg/mL RNAse A for 30 min before FACS. Cytometry was performed using the cell analyzer BD FACSCantoIITM Cell Analyzer (BD Biosciences) with FACS Diva software. FlowJo software was used for further analysis. At least 20,000 cells were analyzed per sample from three independent experiments.
Immunofluorescence (IF): Glass coverslips were pre-treated with 10 µg/mL human FN (Corning) at 4 °C overnight in 24-well plates. Cells were seeded on coverslips at a cell confluence of 70%. After 24 h incubation, cells were washed twice with PBS. Cell fixation was performed with 15 min incubation of 3.7% paraformaldehyde (PFA). PFA was removed, and possible remaining free aldehyde was quenched by additional 15 min incubation with 100 mM glycine. Cells were washed once with PBS and permeabilized by 0.1%-Triton X-100 PBS for 30 min at room temperature. Cells were washed twice with 0.2%-BSA PBS, and samples were prepared for incubating with primary antibodies. Free primary antibody was removed, and cells were washed twice with 0.2%-BSA PBS. Secondary antibody was added for 1 h. In the case of phalloidin staining (Sigma), no secondary antibody was required, and samples were treated as follows. Samples were washed twice with 0.2%-BSA PBS, and after wash with PBS, they were maintained in milliQ water until mounting. A drop of mounting medium (Dako) was used for coverslip mounting on slides. Images from IF were taken in a confocal microscope Nikon A1R+.
sgMMP14 cell development: Human MMP14 sequence (Gene ID 4323), located at chromosome 14, was used to design single-guide RNAs (sgRNA). The sgRNAs were designed by using Benchling software. Design and cloning were performed as previously described by our group. Briefly, the sgRNA oligo duplexes were cloned into the plasmid CBh-hfCas9-2A-eGFP, kindly provided by Dr. Trond Aasen. The sgRNA oligo duplexes were ligated into the CBh-hfCas9-2A-eGFP vector. The plasmid was digested with Bbs1 to generate compatible ends. Gene editing was confirmed by using EnGen™ Mutation Detection Kit (NEB). Clone validation was assessed by WB. Genomic DNA was extracted, and the edited locus was amplified by PCR. Sanger sequencing was used to determine gene editing. To detect indels, the deconvolution tool CRISPR-ID was used (http://crispid.gbiomed.kuleuven.be, accessed on October 2019).
Sanger sequencing: Sanger sequencing was performed at the Genomics and Sequencing Service from the IBiS using the automatic sequencer Applied Biosystems 3500 Genetic Analyzer (Applied Biosystems, Waltham, MA, USA).
Bioinformatics analysis: performed by the Bioinformatics Facility at IBiS. Briefly, array data were quantile normalized by the Robust Multichip Average (RMA) method using the oligo package from R/Bioconductor. Custom ClariomSHuman_Hs_ENSG CDF files from Brainarray (version 24) were used to avoid unspecific and bad quality probe sets.

## 5. Conclusions

Altogether, our data indicate that the link between ENG and MMP14 is relevant in ES cell spreading by regulating actin dynamics and ECM organization. ES dissemination is, however, a highly complex process involving a vast number of proteins and pathways that are tightly coordinated, thus urging the need to further explore each crucial step of the metastasis cascade.

## Data Availability

Expression arrays data available at GEO (#GSE173154).

## Supplementary Materials

The following supporting information can be downloaded at: https://www.mdpi.com/article/10.3390/ijms23158657/s1. More detail can refer to Refs. [23,26,50,51,52,53].
