# Supplementary material for: Endoglin and MMP14 Contribute to Ewing Sarcoma Spreading by Modulation of Cell–Matrix Interactions
Source: Int J Mol Sci. 2022 Aug 4;23(15):8657. doi: 10.3390/ijms23158657 (PMC9369355; doi:10.3390/ijms23158657)
Supplement: Supplementary file 1 [file ijms-23-08657-s001.zip › ijms-1756018-supplementary.pdf]

Supplementary Material and Methods

Cell line-derived xenograft study: All animal experiments were conducted in accordance with protocols and conditions approved by the European guidelines (EU Directive 2010/63/EU). Mouse models used in this work included *in vivo* proliferation assays in RM82/TC71-derived xenografts. All protocols were approved by the local institution and the *Dirección General de la Producción Agrícola y Ganadera de la Junta de Andalucía* in Spain. Cells were implanted double flank, subcutaneously in nude mice, and tumor growth was measured every 2 days, as previously described [1, 2]. Briefly, 2.5 million-cells (RM82 shENG) or 3 million-cells (TC71 pENG) were resuspended in FBS and antibiotic-free medium and mixed with Matrigel Matrix (Corning) on ice. Mice were anesthetized and cell line-xenografted mouse models were generated by subcutaneous injection of the Matrigel-cell suspension. Animals were injected at double flank: on the right flank, with the engineered model, and on the left flank, with the non-targeting control. Once the tumors reached a measurable volume by external calibration with a caliper, the tumoral bulk was measured weekly. Animals were sacrificed when tumor volume reached 1500 mm^3. Tumor growth differences between the control group and individual conditions at different time points were statistically analyzed by paired two-tailed Student's-*t*-test using GraphPad Prism (version 6.0).

Western blotting (WB): Protein lysis using RIPA buffer, protein quantification by Bradford assay and WB were performed as previously described by our group. [1, 2] Herein, the following antibodies were used: anti-ENG (#ab169545, Abcam), anti-MMP14 (#ab51074, Abcam), anti-GAPDH (#2118, Cell Signaling Technology), anti-HA-Tag (#3724, Cell Signaling Technology), anti-CD63 (#10628D, Invitrogen), anti-CD81 (#10630D, Invitrogen), anti-CD9 (#13174, Cell Signaling Technology), anti-FAK1 (#3285, Cell Signaling Technology), anti-pY397 FAK1 (#3283 Cell Signaling Technology), anti-PKCβ (#9371, Cell Signaling Technology), anti-PKC (pan) pSer660 (βII) (#46809, Cell Signaling Technology), anti-Smad2 (C86F7) (#3122, Cell Signaling Technology), anti-Smad2/3 pSer465/Ser423,Ser467/Ser425 (#8828, Cell Signaling Technology), anti-Smad1 (D59D7) (#6944, Cell Signaling Technology), anti-Smad1/5 pSer463/Ser465 (#9516, Cell Signaling Technology). Quantification was performed by densitometry using ImageJ and normalized to GAPDH.

Immunoprecipitation coupled to mass spectrometry (IP/MS):

Protein sample preparation for IP: 3.75 × 10^6 cells (from RM82 WT and shNT) were seeded on three 200-mm plates each, pre-coated with 1 µg/mL GL. After 48 h of incubation, cells were 80–90% confluent. At that time, samples were washed twice with PBS and scraped with Soft Lysis Buffer (Soft lysis buffer was generated in house: 10mM HEPES pH7,9. 10mM KCL, 0.1mM EDTA, 0.1mM EGTA and miliQ water to 10ml, supplemented with 0.5mM PMSF, 1mM DTT (all from SIGMA) and 0.625% Nonidet NP-40 (IGEPAL)) on ice. After 10 min of incubation with frequent mix-shaking, samples were centrifuged at 13.000rpm for 15 min at 4ºC. Protein concentration was

determined by BCA assay (Thermo Fisher). A volume containing 1 mg protein per sample was used for further analysis.

IP: A KingFisher Duo Prime System (Thermo Fisher Scientific) was used to perform an automatic IP following the next steps. The protein sample was incubated with Protein G Dynabeads (G Mag Sepharose™ Xtra beads, GE Healthcare) and anti-ENG antibody P3D1 (#sc-18838, Santa Cruz BT). In the case of samples used as negative controls, protein extracts were incubated with Protein G Dynabeads and Mouse $IgG_{2\alpha}$. After incubation, Dynabeads were washed twice with Soft Lysis Buffer and three times with PBS. Proteins were digested into peptides with 1 mg trypsin per sample at 37ºC for 4 h in Digestion Buffer (2 M urea, 100 mM Tris-HCl pH 7.5, 1 mM DTT).

Sample preparation for MS: The peptides were purified and salts were discarded by the in-stage tip technique using C18 to retain the peptides.[3] After elution, samples were lyophilised and resuspended in 0.1% trifluoroacetic acid (TFA).

MS analysis*:* Samples were injected in an Orbitrap Fusion™ Lumos™ Tribrid™ mass spectrometer (Thermo Fisher Scientific). Protein identification and quantification were performed by the Label-Free Quantification (LFQ) method using MaxQuant software.

Interaction network analysis: Differences between LFQ values derived from IgG isotype control and anti-ENG antibody samples were analysed statistically by paired two-tailed Student's *t*-test using GraphPad Prism. A composite functional association network was constructed from significantly ENG-enriched

proteins using GeneMANIA (version 3.5.1; human interactions) in Cytoscape (version 3.8.0). For integration with the integrin adhesome, the network was merged and connected with a network of the consensus adhesome. The networks were clustered using the force-directed algorithm in the Prefuse toolkit.

Functional enrichment analyses: GO over-representation analysis was performed using WebGestalt (version 2019). Gene family enrichment analysis was performed using ToppGene (build #31). False discovery rates were determined using the Benjamini–Hochberg method.

Reverse-phase protein array (RPPA) analysis:

Sample preparation. RPPA analysis was performed by the Host and Tumour Profiling Unit microarray services at the Institute of Genetics and Cancer, University of Edinburgh (Edinburgh, UK). Briefly, cells were seeded on substrate-precoated 100-mm plates at a confluence not higher than 60% in order to limit cell-to-cell interactions. After 24 h incubation at 37ºC, cells were washed twice with cold PBS and scraped from the plate in the presence of RPPA lysis buffer on ice. To improve protein yield, cells were transferred into a 1.5-mL tube for occasional vortexing, and incubated for 20–30 min on ice. Afterwards, cell lysates were centrifuged at maximum speed for 10 min. Supernatants were recovered and protein concentration was calculated by BCA assay.

Microarray layout: Samples were adjusted to a final concentration of 1 mg/mL with RPPA lysis buffer. The RPPA lysis buffer was 1% Triton X-100, 50mM

HEPES, pH 7.4, 150mM NaCl, 1.5mM MgCl2, 1mM EGTA, 100mM NaF, 10mM Na pyrophosphate, 1mM Na3VO4 (pre-activated), 10% glycerol, containing freshly added protease and phosphatase inhibitors from Roche Applied Science Cat. # 05056489001 and # 04906837001, respectively. Protein samples were mixed with 0.25 volume of 4× SDS 10% BME (beta-mercaptoethanol) sample buffer without bromophenol blue and denatured at 95ºC for 5 min. Each sample was prepared into 4 serial dilutions (100, 50, 25 and 12.5% from the 1 mg/mL stock sample) with 10%-glycerol PBS in a 96-well plate. A total of 36 samples, with 4 diluted replicates each, were transferred into a 384-well plate. That plate would be the source plate from which samples were dotted on a Supernova Nitrocellulose slide (Grace BioLabs) in technical triplicate per array. Each slide comprised eight arrays. A total of 9 slides were printed. The array spotting was carried out with an Aushon 2740 Arrayer Platform using 185-µm pins, which deposited sample dots with 500 µm distance from spot to spot.

RPPA assay: Eight printed slides were washed with deionised water for 15 min four times. Slides were incubated with antigen retrieval reagent (1× ReBlot Strong, ReBlot Plus Strong Antibody Stripping Solution, #2504 Millipore) for 10 min. After two washes of deionised water for 5 min, the slides were placed in ProPlate Multi-Well Chambers, #470639 Grace Bio-Labs, where each well of the chamber would correspond to a single array. Wells were filled with deionised water. Within the chamber, slides were washed twice with 1× TBS-T (0.05 M Tris-HCl, 0.3 M NaCl with 0.1% Tween-20) for 5 min. SuperBlock^{TM} T20 (TBS) (Thermo Fisher Scientific) was used as a blocking buffer for 10 min. After two washes with TBS-T for 5 min, a primary antibody diluted 1:250 in

Superblock was added per chamber. A total of 60 antibodies were used, including rabbit and mouse secondary antibodies that were considered as off-target controls. Slides were washed twice with TBS-T for 5 min. Another blocking with TBS was performed for 10 min. After two washes with TBST for 5 min, slides were incubated with a secondary antibody (DyLight^{TM} 800-labeled anti-species antibody, Invitrogen) diluted 1:2500 in TBS for 30 min. Slides were washed twice with TBS-T for 5 min. Finally, slides were rinsed with deionised water and were protected from light at room temperature to dry, for 10 min. For protein normalisation, a slide was stained with FAST Green (Grace^{TM} Bio-Labs), which is a protein dye used to quantify protein samples loaded on arrays. After printing, a slide was washed with distilled water for 5 min with agitation and was incubated with 1% NaOH for 5 min. Repeated brief rinses of the slide under distilled water over 1 minute was followed by a 5-min wash in agitation. The slide was incubated with FAST Green Staining solution for 3 min in agitation. Repeated brief rinses of the slide under distilled water over 1 min was followed by a 15-min wash with De-Staining Solution (30% methanol and 7% glacial acetic acid) in agitation. The slide was washed by repeatedly rinsing under distilled water over 1 min. Centrifugation was finally used to dry the slide.

Data acquisition and analysis*:* Slides were scanned using an Innopsys 710 slide scanner (Innopsys). Microarray images were analysed by Mapix software (Innopsys). A spot grid adjusted to the dot distribution along the array was designed to measure the fluorescence intensity. The spot diameter in the grid was set to 270 µm. Mean background from the adjacent area surrounding each spot was subtracted from the absolute fluorescent signal. Quality control of the

technique used the linear fit of the 4-dilution series of each sample, where R^2 > 0.9 was deemed good, R^2 > 0.8 was deemed acceptable, and R^2 < 0.8 was deemed poor and was excluded from data analysis. The Relative Fluorescence Intensity (RFI) was calculated as the median value from the 4-point dilution series. A final normalisation with FastGreen sample spots was performed, and the mean of the three technical replicates was used as net RFI.

sgMMP14 cell development: Human *MMP14* sequence (Gene ID 4323), located at chromosome 14, was used to design single guide RNAs (sgRNA). The sgRNAs were designed by using Benchling software. Design and cloning were performed as previously described by our group. Briefly, the sgRNA oligo duplexes were cloned into the plasmid CBh-hfCas9-2A-eGFP, kindly provided by Dr. Trond Aasen. The sgRNA oligo duplexes were ligated into the CBh-hfCas9-2A-eGFP vector. The plasmid was digested with Bbs1 to generate compatible ends. Gene editing was confirmed by using EnGen™ Mutation Detection Kit (NEB). Clone validation was assessed by WB. Genomic DNA was extracted and the edited locus was amplified by PCR. Sanger sequencing was used to determine gene editing. To detect indels, the deconvolution tool CRISPR-ID was used (http://crispid.gbiomed.kuleuven.be).

Sanger sequencing: Sanger sequencing was performed at the Genomics and Sequencing Service from the IBiS using the automatic sequencer Applied Biosystems 3500 Genetic Analyzer (Applied Biosystems).

Bioinformatics analysis*:* Bioinformatic analysis was performed by the Bioinformatic Facility at IBiS. Briefly, array data were quantile normalized by the Robust Multichip Average (RMA) method using the oligo package from R/Bioconductor. Custom ClariomSHuman_Hs_ENSG CDF files from Brainarray (version 24) were used to avoid unspecific and bad quality probe sets.

qRT-PCR: Total RNA from the cell lines was extracted by using the miRNeasy kit (QIAGEN) and qRT-PCR was performed as previously described by our group.[1, 2] TaqMan probes were used to determine gene expression of *GAPDH*, *ENG*, *MMP14*, *ACVR2A, ACVR2B, ACVRL1, BMPR1A, BMPR1B, TGFBR1, TGFBR2, NID1, ANXA2, PALLD, MMP16, FN1, PHLDB2*. Data from qRT-PCR studies were analysed with S.D.S. (Sequence Detection System version 2.4). The formula $2^{-\Delta Ct}$ was used, as previously described by us.[1, 2] Differences between mRNA levels from the control group and individual conditions were analysed statistically by paired two-tailed Student's *t*-test using GraphPad Prism**.**

Expression arrays: RNA integrity was confirmed by a bioanalyzer (Agilent, RNA Nano 6000) and the human transcriptome was evaluated using the Clariom^{TM} S Human array (ThermoFisher) by the Genomic Facility at IBiS, following manufacturer's instructions. RNA was amplified and labeled using the GeneChip® WT PLUS Reagent Kit (Thermo Fisher Scientific, Inc.) Amplification was performed with 100 ng of total RNA input following procedures described in the WT PLUS Reagent Kit user manual. The amplified cDNA was quantified, fragmented, and labeled in preparation for hybridization to GeneChip® Clariom S Human Array (Thermo Fisher Scientific, Inc.) using 5.5 µg of single-stranded cDNA product and following protocols outlined in the user manual. Washing,

staining (GeneChip® Fluidics Station 450, Thermo Fisher Scientific, Inc.), and scanning (GeneChip® Scanner 3000, Thermo Fisher Scientific, Inc.) were performed following protocols outlined in the user manual for cartridge arrays. Data available at Gene Expression Omnibus (GSE173154).

Lentiviral transduction:

HEK293T transfection: The day before transfection, 3 × 10^6 HEK293T cells were seeded on a 100-mm plate. HEK 293T cells were grown in DMEM 10%FBS and 1%P/S 1%glutamine. The next day, when HEK293T cells were at 80–90% confluence, plasmid DNA with transfection Polyplus.

Transduction on target cells: Target cells were seeded on 100-mm plates. Supernatant from transfected HEK293T cells was collected and centrifuged at 1800 rpm to discard dead cells. Cells were cultured with filtered HEK293T supernatant, which was previously mixed with 4 mg/mL polybrene (Sigma). After 24 h, the supernatant was discarded. At 48 h of viral particle incubation, antibiotic selection started (pLKO- puromycin; pDisplay-G418). Antibiotic pressure was maintained until individual clones were selected on the plate.

MTT assay: MTT assay (Roche) was used to evaluate cell proliferation, under manufacturer's instructions and as previously described by our group. [1] Absorbance of the colorimetric reaction was measured at 565 nm by using a microplate reader (TECAN). Differences between the control group and individual conditions were analysed statistically by paired two-tailed Student's *t*-test using GraphPad Prism**.**

Clonogenicity assay: Cells were seeded at a low confluence on 6-well plates, 500 cells per condition After colony formation (day 10 for RM82 and TC71, day 14 for SKNMC), cells were washed with PBS, fixed at room temperature. Wells were washed three times with PBS and colonies were stained with crystal violet, washed and individual clones were counted. Differences between the control group and individual conditions were analysed statistically by paired two-tailed Student's *t*-test using GraphPad Prism.

Migration assay: The Cultrex® 2- well BME cell invasion assay (Trevigen) was used to measure cell chemotactic migration, under manufacturer's instructions and as previously described by our group. Buffer containing calcein-AM (# C1430, ThermoFisher) was used to stain live cell and fluorescence of the solution from the bottom chamber was measured at 485 nm excitation and 520 nm emission by using a microplate reader (TECAN). Differences between the control group and individual conditions were analysed statistically by paired two-tailed Student's t-test using GraphPad Prism.

Attachment assay: Attachment assays were performed and adapted according to the standardized method described by Martin J. Humprhies. [4] 96-well plates were pre-coated with 10 µg/mL FN (Corning) overnight at 4ºC. Cells were deprived of serum for 12 h before the assay. Wells were washed twice with cold PBS. The bottom of the wells was blocked by incubation with a Blocking Buffer (PBSwith 2%BSA) for 1 h at 37ºC. Cells were seeded in confluence of 2·104 cells/well on a 96-well plate and incubated for 15 min at 37ºC. Supernatant was gently aspirated and wells were washed once with cold PBS to remove non-adherent cells. After washing, cells were incubated with 10% FBS medium for 4 h at 37ºC for cell recovery. The number of adhered cells was estimated using MTT assay. Differences between the control group and individual conditions were analysed statistically by paired two-tailed Student’s t-test using GraphPad Prism.

Spreading assay: Cells were incubated longer than 24 h seeded on the plate to confirm they were actively growing at the time of the experiment. 96-well plates were pre-treated with 20 µg/mL FN overnight at 4ºC, or 1 h at 37ºC. Wells were aspirated and washed with Wash Buffer (cold PBS) twice. Subsequently, wells were incubated with Blocking Buffer (standard blocking solution of 2% BSA in PBS) at 37ºC for 45–60 min. During that time, a solution of cells was prepared in medium (RPMI)/HEPES. Plates were washed with Wash Buffer. 2×10^4 cells/well suspension were incubated for 1 h at 37ºC. Afterwards, cells were fixed with 50% (w/v) glutaraldehyde. The fixative medium was aspirated and fixed cells were maintained in 0.05% NaN_3. Cell spreading was assessed by imaging acquisition in a direct microscope Olympus BX-71. Differences between the control group and individual conditions were analysed statistically by paired two-tailed Student's *t*-test using GraphPad Prism.

Actin filament and filopodia analysis*:* Images from phalloidin-stained cells were obtained under a confocal microscope Nikon A1R+ after performing IF. The number of cells was identified by DAPI staining. Fluorescent cell area from different 40× fields at 60–70% cell confluence was measured with ImageJ. Filopodia analysis was performed using the FiloQuant plugin from Fiji (Single Image FiloQuant version was used). FiloQuant setting values were as follows: (a) Edge detection: (a.1) Threshold for cell edges: 3; (a.2) Number of iterations for Open: 10; (a.3) Number of iterations for Erode: 0; (a.4) Fill holes on edge: no; (a.5) Fill holes: no; (b) Filopodia detection: (b.1) Threshold for filopodia: 25; (b.2) Filopodia minimum size: 25; (b.3) Use convolve to improve filopodia detection: yes; (b.4) Use focal contrast enhancement to improve filopodia detection: yes; (c) Contour detection: (c.1) Number of iterations for Close: 4; (c.2) Number of iterations for Erode: 4; (c.3) Number of iterations for Dilate: 4. More than 30–50 cells were studied by condition in duplicates in 3 independent experiments. Differences between the control group and individual conditions were analysed statistically by paired two-tailed Student’s t-test using GraphPad Prism.

*Extracellular Vesicles (EV) isolation by Ultracentrifugation (UC):*Cells were seeded in 10 175-mL flasks with 10% FBS 1% P/S medium. When cells reached 70–80% confluence, flasks were washed three times with EV-free filtered PBS (Corning® 500 mL Vacuum Filter/Storage Bottle System, 0.22 µm Pore 33.2cm² PES Membrane #431097 Corning). An EV-depleted medium (Filtered medium supplemented with 10% exosome-free-FBS Exo-FBS, systembio) was used for 24 h incubation. Supernatant was collected after 24–48 h, when cells were 90% confluent. Tubes were centrifuged 300 *g* for 10 min at room temperature to pellet dead cells. Samples were centrifuged in Evolution RC6 PLUS centrifuge with fixed rotor at 4000 rpm for 30 min at 4ºC. Supernatants were collected into polycarbonate tubes and samples were centrifuged in a OPTIMA L100 XP centrifuge with Sw32Ti rotor at 110,000 *g* for 2 h at 4ºC. Supernatant was discarded and the resulting pellet was diluted and centrifuged in OPTIMA L100 XP with Sw32Ti rotor at 110000 *g* for 2 h at 4ºC. Supernatant was, again, discarded and pellets containing EVs were incubated with RIPA buffer for at least 20 min on ice for protein extraction[5]

EV isolation: OptiPrep^{TM} Density Gradient (ODG) isolation:

Sample concentration: Samples were thawed overnight at 4ºC. Samples were placed in polycarbonate tubes and centrifuged at 100,000 *g* for 3h 30 min at 4ºC in an OPTIMA L100 XP centrifuge with a Sw32Ti rotor. ODG preparation was performed as described elsewhere.[6] The gradient was pipetted with a 1 mL pipette very slowly and carefully for appropriate gradient separation. The sample (1 mL PBS-resuspended) was layered on top of the gradient. Samples were centrifuged in a OPTIMA L100 XP centrifuge for 18 h at 4ºC.

Fraction collection: 1 mL fractions of the gradient were placed in 1.5 mL tubes separately. F1 corresponded to the top fraction, whereas F16 was the bottom

fraction. Tubes were weighed before grouping them (F1; F2-3-4; F5-6-7; F8-9-10; F11-12-13; F14-15-16) in centrifuge tubes except F1.

Fraction preparation for protein evaluation*:* Fractions were resuspended in EV-depleted PBS until a final volume of 12 mL each tube. Samples were centrifuged at 100,000 *g* for 180 min at 4ºC to remove OptiPrep^{TM}. Supernatant was discarded and pellets were incubated with a RIPA lysis buffer for 20 min and kept at −20ºC until protein quantification.

IHC*:*At the end of the *in vivo* experiments, animals were sacrificed in CO_2 chambers and tumours were collected from the animal flank. Representative parts of the tumour samples were snap-frozen or fixed in formol for 24 h and paraffin-embedded. IHC was performed at HUVR Biobank facilities and the following antibodies were tested: anti-ENG (#ab170943, Abcam) and anti-MMP14 (#ab51074, Abcam). Intensity and area of staining from the antibody staining was evaluated by an experienced pathologist, where the null score was deemed 0 and the highest score was deemed 3.

Supplementary Information

Figure S1. ENG and MMP14 in ES patients and *in vitro* models. (A) Kaplan Meyer of OS of ES patients in groups according to High and Low ENG and/or MMP14 expression- (p<0,001). **(B)** Positive mRNA correlation between the normalized mRNA gene expression of ENG and MMP14 (R2=0,064,p<0,001) **(C,D)** Generation of knockdown models of ENG in the RM82 **(D)** and SK-NMC **(D)** cell lines. Candidate clones were confirmed by qRT-PCR. **(E)** FACS analysis depicting a decrease on the number of cells expressing transmembrane ENG in the downregulated models as well as **(F)** a decrease in the median fluorescent intensity (MFI).

Figure S2. Working pipeline and generation of the engineered models: transcriptomics and proteomics analysis: downregulated models in green, upregulated models in red, proteomics in blue.

Figure S3. ENG impact in self-renewal and 3D-dependent proliferation *in vitro.* **(A,B)** Clonogenic assay performed in RM82- from **(A),** SK-N-MC- **(B)** sheng models and the **(C)** TC71-pENG upregulated model. The downregulation of ENG significantly affected clonogenicity, on contrast to the upregulation of ENG. Representative images. **(D)** Two different cell densities were seed in Ultra-low-adherence plates to confirm that ENG was not involved in cell-cell adhesion. Representative images**.** **(E)** No ENG-dependent differences were observed regarding the cell cycle profile of the RM82 shENG model. **(F)** *In vitro* 2D proliferation assay in shENG model from SK-N-MC. No significant differences were observed between the ENG downregulated conditions and the NT control. **(G)** 3D proliferation assay determined an increased tumour growth in the absence of ENG. **(H,I)** ENG expression was validated in the RM82-shENG **(H)** and in the TC71-pENG **(I)** xenografted models *in vivo.* Paired two-tailed Student *t*-test was performed to evaluate statistical difference among groups, where ns: non-significant; *$p < 0.05$; **$p < 0.001$ and ***$p < 0.0001$.

Figure S4. Evaluation of TGFβ receptors (ACVR2A, ACVR2B, ACVRL1, BMPR1A, BMPR1B, TGFBR1 and TGFBR2) by qRT-PCR determined low or null expression of the genes in a panel of ES cell lines (n = 8).

Figure S5. Validation of the transcriptomic analysis of RM82-shENG and TC71-pENG models. **(A)** Transcriptomic data from RM82-shENG and TC71-pENG were analysed by GSEA. Inversely common GO terms and gene signatures are depicted in the heatplots. **(B,C)** Genes deferentially expressed were validated by qRT-PCR in the RM82-shENG **(B)** and TC71-pENG **(C)** models. Three biological replicates were used. Paired two-tailed Student *t*-test was performed to evaluate statistical difference among groups, where ns: non-significant; *$p < 0.05$; **$p < 0.001$ and ***$p < 0.0001$.

Figure S6. ENG and MMP14 expression are not regulated by EWS-FLI1-independent manner. (A) *LOX* was used as a control gene regulated by the fusion protein. FPKM results on *ENG* and *MMP14* revealed a similar gene modulation, where the expression may not be dependent on the expression of *EWSR1-FLI1*. Adapted from *Tomazou et al.* 2015. **(B)** Quantification of ENG and MMP14 expression determined by WB in the presence and absence of EWS-FLI1 without detecting any significant difference at the protein level.

Figure S7. Validation of the transcriptomic analysis of RM82-sgMMP14. **(A)** Genes differentially expressed according to the microarray data were validated by qRT-PCR in the RM82-sgMMP14 model. Three biological replicates were used. Paired two-tailed Student t test was performed to evaluate statistical difference among groups, where ns: non-significant; *$p < 0.05$; **$p < 0.001$ and ***$p < 0.0001$ and ****$p < 0.00001$.

Figure S8. ENG regulates cell-substrate adhesion in ES cells and is enriched in EVs. (A) The evaluation of cell spreading was performed by seeding pENG cells on 20 µg/mL FN for 1 h. Cell area, highlighted in yellow, was used as a reference for spreading. **(B)** Different cell spreading in cells overexpressing ENG with respect to the control group associated with higher mean areas. **(C)** UC-isolated EVs from the RM82 cell line presented enrichment in tetraspanins (CD63, CD81 and CD9). Enrichment in ENG was also confirmed by WB. **(D)** Alternatively, EVs were isolated by OptiPrep density gradient and confirmed by fraction density. **(E)** As determined by WB, ODG-isolated EVs from the RM82 cell line were identified in different fractions enriched in CD63, CD81 and CD9, which also showed enrichment of ENG.

Figure S9. ENG contributes to actin distribution in ES cells. (A) Cell aggregates were observed in shENG cells seeded on 20 µg/mL FN, in contrast to the control condition. **(B)** Cell density of the confocal fields evaluated presented a similar number of cells in all groups except for shENG2, with a higher cell density. **(C)** Filopodia length was measured by Fiji (FiloQuant) and determined an ENG-dependent effect on RM82 shENG cells. **(D)** IF of actin

filaments stained by Phalloidin-TRITC (grey) in the TC71-pENG model. Three biological replicates were performed for each experiment. Photos are a representative image of each experiment.

Figure S10. Loss of ENG affects the distribution of actin filaments. Z-stack superposition images from shNT and RM82-shENG cells, confirmed the ENG-dependent filamentous actin organization. White arrows signal actin filaments.

Figure S11. Loss of ENG results in accumulation of actin aggregates. Z-stack superposition images from WT, shNT and shENG RM82 cells, respectively, confirmed the regulation of ENG on the actin filaments assembly, and the accumulation of actin aggregates.

Figure S12. Downregulation of protein phosphorylation and expression in an ENG-dependent manner. (A,B) Phosphorylation of Y397 FAK1 **(A)** and S660 PKCβII **(B)** was impaired in shENG seeded on GL, FN and PDL. **(C)** Phosphorylation of S217,221 PLCγ was downregulated in shENG seeded on PDL. **(D)** β-actin levels were lower in shENG cells seeded on FN. **(E,F)** Phosphorylation of MEK1/2 in RM82-shENG cells seeded on GL and PDL **(E)** and phosphorylation of ERK on cells seeded on GL, FN and PDL **(F)** were affected. **(G,H)** No significant changes were observed regarding CAV1 and ITGα4. Graphs depict individual analysis of each protein, derived from the RRPA study. RFI stands Relative Fluorescence Intensity. Statistical significance was evaluated by paired two-tailed Student *t*-test, where ns: non-significance, *$p < 0.05$; **$p < 0.001$ and ****$p < 0.00001$.

Figure S13. Upregulation of signalling pathways upon downregulation of ENG. (A) The phosphorylation of T419 and T403 PKCλ/ζ was increased upon ENG downregulation. **(B,C)** shENG cells presented an upregulated phosphorylation of S473 (**B)** and S308 **(C)** AKT when seeded on GL, FN and PDL**. (D)** Upregulation of S2481 mTOR was observed in the downregulation of ENG, when cell were seeded on GL. **(E,F)** No differences on SMAD1/5 phosphorylation were observed **(E)** Only upregulation of SMAD2/3 phosphorylation in one clone was observed when cells were seeded on GL and PDL **(F)**. Graphs depict individual analysis of each protein, derived from the RRPA study. RFI stands Relative Fluorescence Intensity. Paired two-tailed Student *t*-test was performed to evaluate the statistical significance, where ns: non-significance, $*p < 0.05$; $**p < 0.001$; $***p < 0.001$ and $****p < 0.00001$.

Figure S14. Substrate-dependent regulation of ES signalling pathways related to ENG. (A,B) Phosphorylation of Y397 FAK1 **(A)** and Y416 Src **(B)** were reduced in cells seeded on PDL in comparison to FN. **(C)** Endogenous levels of Src were upregulated in cells seeded on PDL. **(D,E)** Phosphorylation of S2448 **(D)** and S2481 **(E)** mTOR were reduced in cells seeded on PDL in comparison to the phosphorylation induced on FN. Graphs depict individual analysis of each protein, derived from the RRPA study. RFI stands Relative Fluorescence Intensity. Statistical significance was addressed by paired two-tailed Student t-test, where ns: non-significance, $*p < 0.05$; $**p < 0.001$; $***p < 0.001$ and $****p < 0.00001$.

Figure S15. Validation of RPPA PKC results. (A,B) Quantification of endogenous expression of PKCβ by WB. **(C,D)** Quantification of the phosphorylated S660 PKCβ normalized to endogenous expression of PKCβ, by WB. (a.u. stands for arbitrary units) Statistical significance was addressed by paired two-tailed Student *t*-test, where ns: non-significance, $*p < 0.05$.

Figure S16. IP/MS identifies ENG associates with the dynactin complex within the global network. (A) Dynactin family members were highly enriched and formed an interconnected cluster in the ENG interaction network. **(B)** Detail of global network interaction. (Network edges (connecting lines) represent reported physical (red), genetic (green), pathway (blue) or predicted (dashed orange) interactions or imputed interactions detected by IP/MS (dashed gray). Nodes (circles) represent identified proteins. Node size is proportional to log_2-transformed LFQ ratio (ENG/IgG); node fill color represent *p*-value of LFQ ratio (log_{10} scale).

Figure S17. Representative model based on our data, displaying ENG and MMP14 ECM modulation and effects on cell migration.

1. Puerto-Camacho, P.; Amaral, A. T.; Lamhamedi-Cherradi, S. E.; Menegaz, B. A.; Castillo-Ecija, H.; Ordonez, J. L.; Dominguez-Hormaetxe, S.; Jordan-Perez, C.; Diaz-Martin, J.; Romero-Perez, L.; Lopez-Alvarez, M.; Civantos-Jubera, G.; Robles-Frias, M. J.; Biscuola, M.; Ferrer, C.; Mora, J.; Cuglievan, B.; Schadler, K. L.; Seifert, O.; Kontermann, R. E.; Pfizenmaier, K.; Simon, L.; Fabre, M.; Carcaboso, A. M.; Ludwig, J. A.; de Alava, E., Preclinical efficacy of endoglin-targeting antibody-drug conjugates for the treatment of Ewing sarcoma. *Clinical cancer research : an official journal of the American Association for Cancer Research* **2018**.

2. Rodriguez-Nunez, P.; Romero-Perez, L.; Amaral, A. T.; Puerto-Camacho, P.; Jordan, C.; Marcilla, D.; Grunewald, T. G.; Alonso, J.; de Alava, E.; Diaz-Martin, J., Hippo pathway effectors YAP1/TAZ induce an EWS-FLI1-opposing gene signature and associate with disease progression in Ewing sarcoma. *J Pathol* **2020,** 250, (4), 374-386.
3. Turriziani, B.; Garcia-Munoz, A.; Pilkington, R.; Raso, C.; Kolch, W.; von Kriegsheim, A., On-beads digestion in conjunction with data-dependent mass spectrometry: a shortcut to quantitative and dynamic interaction proteomics. *Biology* **2014,** 3, (2), 320-32.
4. Humphries, M. J., Cell adhesion assays. *Methods in molecular biology* **2009,** 522, 203-10.
5. Peinado, H.; Aleckovic, M.; Lavotshkin, S.; Matei, I.; Costa-Silva, B.; Moreno-Bueno, G.; Hergueta-Redondo, M.; Williams, C.; Garcia-Santos, G.; Ghajar, C.; Nitadori-Hoshino, A.; Hoffman, C.; Badal, K.; Garcia, B. A.; Callahan, M. K.; Yuan, J.; Martins, V. R.; Skog, J.; Kaplan, R. N.; Brady, M. S.; Wolchok, J. D.; Chapman, P. B.; Kang, Y.; Bromberg, J.; Lyden, D., Melanoma exosomes educate bone marrow progenitor cells toward a pro-metastatic phenotype through MET. *Nature medicine* **2012,** 18, (6), 883-91.
6. Van Deun, J.; Mestdagh, P.; Sormunen, R.; Cocquyt, V.; Vermaelen, K.; Vandesompele, J.; Bracke, M.; De Wever, O.; Hendrix, A., The impact of disparate isolation methods for extracellular vesicles on downstream RNA profiling. *Journal of extracellular vesicles* **2014,** 3.
