# Supplementary material for: Endoglin and MMP14 Contribute to Ewing Sarcoma Spreading by Modulation of Cell–Matrix Interactions
Source: Int J Mol Sci. 2022 Aug 4;23(15):8657. doi: 10.3390/ijms23158657 (PMC9369355; doi:10.3390/ijms23158657)
Supplement: Supplementary file 1 [file ijms-23-08657-s001.zip › Tables S1-S8.pdf]

A ENG		Primary			Post-CT			Metastasis			Relapse		
**Intensity**	**Score**	**# scored**	**% total per group**		**# scored**	**% total per group**		**# scored**	**% total per group**		**# scored**	**% total per group**	
**Negative**	**0**	1	2.86	22.86	6	26.09	60.87	0	0.00	31.25	1	12.50	37.50
**Low**	**(0,1]**	7	20.00		8	34.78		5	31.25		2	25.00	
**Intermediate**	**(1,2]**	17	48.57	71.14	6	26.09	39.13	9	56.25	68.75	2	25.00	62.50
**High**	**(2,3]**	10	28.57		3	13.04		2	12.50		3	37.50	
	**Total**	35	100	100	23	100	100	16	100	100	8	100	100

B ENG		Primary			Post-CT			Metastasis			Relapse		
**IxA**	**Score**	**# scored**	**% total per group**		**# scored**	**% total per group**		**# scored**	**% total per group**		**# scored**	**% total per group**	
**Negative**	**0**	1	2.86	28.57	6	26.09	65.22	1	6.25	31.25	1	12.50	37.50
**Low**	**(0,3]**	9	25.71		9	39.13		4	25.00		2	25.00	
**Intermediate**	**(3,6]**	17	48.57	71.43	5	21.74	34.78	9	56.25	68.75	2	25.00	62.50
**High**	**(3,9]**	8	22.86		3	13.04		2	12.50		3	37.50	
	**Total**	35	100	100	23	100	100	16	100	100	8	100	100

C ENG		# scored							
**Intensity**	**Score**	**Primary**	**% All total**	**Post-CT**	**% All total**	**Metastasis**	**% All total**	**Relapse**	**% All total**
**Negative**	**0**	1	9.8	6	17.1	0	6.1	1	3.7
**Low**	**(0,1]**	7		8		5		2	
**Intermediate**	**(1,2]**	17	32.9	6	11.0	9	13.4	2	6.1
**High**	**(2,3]**	10		3		2		3	
	**Total**	35	42.7	23	28.0	16	19.5	8	9.8

Table S1. Distribution of ENG intensity in ES patients. (A) Frequency of ENG intensity in IHC samples from ES patients. **(B)** Frequency of ENG intensity considering area of staining in IHC samples from ES patients. **(C)** Frequency of ENG intensity considering the frequency of each stage as part of a whole group.

A	MMP14	Primary			Post-CT			Metastasis			Relapse		
**Intensity**	**Score**	**# scored**	**% total**	**% total per group**	**# scored**	**% total**	**% total per group**	**# scored**	**% total**	**% total per group**	**# scored**	**% total**	**% total per group**
**Negative**	**0**	1	2,86	20.00	4	17.39	60.87	2	12.50	37.50	1	12.50	62.50
**Low**	**(0,1]**	6	17.14		10	43.48		4	25.00		4	50.00	
**Intermediate**	**(1,2]**	22	62.86	80.00	6	26.09	39.13	5	31.25	62.50	2	25.00	37.50
**High**	**(2,3]**	6	17.14		3	13.04		5	31.25		1	12.50	
	Total	35	100	100	23	100	100	16	100	100	8	100	100

B	MMP14	Primary			Post-CT			Metastasis			Relapse		
**IxA**	**Score**	**# scored**	**% total**	**% total per group**	**# scored**	**% total**	**% total per group**	**# scored**	**% total**	**% total per group**	**# scored**	**% total**	**% total per group**
**Negative**	**0**	1	2.86	22.86	5	21.74	65.22	2	12.50	43.75	1	12.50	62.50
**Low**	**(0,3]**	7	20.00		10	43.48		5	31.25		4	50.00	
**Intermediate**	**(3,6]**	21	60.00	77.14	5	21.74	34.78	4	25.00	56.25	2	25.00	37.50
**High**	**(3,9]**	6	17.14		3	13.04		5	31.25		1	12.50	
	**Total**	35	100	100	23	100	100	16	100	100	8	100	100

C	MMP14	# scored							
**Intensity**	**Score**	**Primary**	**% All total**	**Post-CT**	**% All total**	**Metastasis**	**% All total**	**Relapse**	**% All total**
**Negative**	**0**	1	8.5	4	17.1	2	7.3	1	6.1
**Low**	**(0,1]**	6		10		4		4	
**Intermediate**	**(1,2]**	22	34.1	6	11.0	5	12.2	2	3.7
**High**	**(2,3]**	6		3		5		1	
	**Total**	35	42.7	23	28.0	16	19.5	8	9.8

Table S2. Distribution of MMP14 intensity in ES patients. **(A)** Frequency of MMP14 intensity in IHC samples from ES patients. **(B)** Frequency of MMP14 intensity considering area of staining in IHC samples from ES patients. **(C)** Frequency of MMP14 intensity considering the frequency of each stage as part of a whole group.

shENG	Database Molecular Signature	NES	pvalue	p.adjust	qvalues
RM82	GO_EXTRACELLULAR_MATRIX	-1,56	1,49E-03	1,32E-01	1,23E-01
SKNMC		-2,10	2,04E-03	6,76E-02	6,08E-02
RM82	GO_REGULATION_OF_TRANSMEMBRANE_TRANSPORT	-1,45	1,49E-03	1,32E-01	1,23E-01
SKNMC		1,34	1,58E-02	2,24E-01	2,01E-01
RM82	GO_SIDE_OF_MEMBRANE	-1,46	1,51E-03	1,32E-01	1,23E-01
SKNMC		-1,35	6,00E-03	1,40E-01	1,26E-01
RM82	GO_REGULATION_OF_TRANS_SYNAPTIC_SIGNALING	-1,38	1,51E-03	1,32E-01	1,23E-01
SKNMC		-1,28	1,61E-02	2,24E-01	2,02E-01
RM82	GO_SYNAPTIC_MEMBRANE	-1,53	1,51E-03	1,32E-01	1,23E-01
SKNMC		-1,43	4,07E-03	1,10E-01	9,93E-02
RM82	BOQUEST_STEM_CELL_CULTURED_VS_FRESH_UP	-1,46	1,51E-03	1,32E-01	1,23E-01
SKNMC		-1,33	1,20E-02	1,97E-01	1,77E-01
RM82	GO_EXTRACELLULAR_STRUCTURE_ORGANIZATION	-1,60	1,52E-03	1,32E-01	1,23E-01
SKNMC		-2,24	2,00E-03	6,76E-02	6,08E-02
RM82	GO_SYNAPSE_ORGANIZATION	-1,43	1,54E-03	1,32E-01	1,23E-01
SKNMC		-1,78	2,04E-03	6,76E-02	6,08E-02
RM82	GO_CELL_SUBSTRATE_JUNCTION	-1,57	1,54E-03	1,32E-01	1,23E-01
SKNMC		-1,52	2,06E-03	6,76E-02	6,08E-02
RM82	BOQUEST_STEM_CELL_UP	-1,88	1,54E-03	1,32E-01	1,23E-01
SKNMC		-2,06	2,05E-03	6,76E-02	6,08E-02

Table S3. GSEA from RM82- and SKNMC- shENG models.

Cell line shENG/pENG	Database Molecular Signature	NES	p value	p adjust	q value
RM82	RIGGI_EWING_SARCOMA_PROGENITOR_DN	-1,43	1,03E-02	2,52E-01	2,33E-01
TC71		1,35	2,00E-02	1,43E-01	1,19E-01
RM82	GO_ACTOMYOSIN	-1,72	1,80E-03	1,88E-01	1,75E-01
TC71		1,45	1,82E-02	1,33E-01	1,11E-01
RM82	GO_COLLAGEN_BINDING	-1,54	9,06E-03	2,45E-01	2,26E-01
TC71		1,53	2,07E-02	1,45E-01	1,21E-01
RM82	GO_CELL_SUBSTRATE_ADHESION	-1,30	3,04E-02	3,53E-01	3,27E-01
TC71		1,42	1,95E-03	3,15E-02	2,63E-02
RM82	GO_CELL_SUBSTRATE_JUNCTION	-1,66	1,58E-03	1,88E-01	1,75E-01
TC71		1,72	1,93E-03	3,15E-02	2,63E-02
RM82	GO_CELL_LEADING_EDGE	-1,45	1,58E-03	1,88E-01	1,75E-01
TC71		1,56	1,92E-03	3,15E-02	2,63E-02

Table S4. GSEA from RM82-shENG and TC71- pENG models.

Subpathway_name	log2(FC)	Pvalue	FDR	UniprotKB_functions
Leukocyte transendothelial migration: PXN	-2,03E-01	9,70E-07	2,42E-04	Cell adhesion
Ras signaling pathway: MLLT4*	1,50E-01	1,10E-06	2,42E-04	Cell adhesion
Ras signaling pathway: MLLT4	1,40E-01	1,71E-06	2,69E-04	Cell adhesion
Sphingolipid signaling pathway: PRKCA	-1,79E-01	1,70E-05	1,06E-03	Apoptosis, Angiogenesis, Cell adhesion, Transcription regulation, Transcription, Adaptive immunity, Immunity, Biological rhythms
Tight junction: TJP1 CTNNB1 CTNNA1	-4,60E-02	1,73E-05	1,06E-03	Cell adhesion, Neurogenesis, Wnt signaling pathway, Transcription regulation, Transcription, Differentiation
Wnt signaling pathway: PRKCA	-1,66E-01	2,22E-05	1,29E-03	Apoptosis, Angiogenesis, Cell adhesion, Transcription regulation, Transcription, Adaptive immunity, Immunity, Biological rhythms
Melanogenesis: PRKACA	-1,84E-01	3,11E-05	1,63E-03	Angiogenesis, Cell adhesion, Apoptosis
Natural killer cell mediated cytotoxicity: PRKCA	-1,59E-01	7,74E-05	2,75E-03	Apoptosis, Cell adhesion, Angiogenesis, Adaptive immunity, Immunity, Transcription regulation, Transcription, Biological rhythms
Retrograde endocannabinoid signaling: PRKCA	-1,62E-01	7,78E-05	2,75E-03	Apoptosis, Angiogenesis, Cell adhesion, Adaptive immunity, Transcription regulation, Transcription, Immunity, Biological rhythms
Cholinergic synapse: PRKCA	-1,62E-01	7,78E-05	2,75E-03	Apoptosis, Angiogenesis, Cell adhesion, Transcription regulation, Transcription, Adaptive

Subpathway_name	log2(FC)	Pvalue	FDR	UniprotKB_functions
				immunity, Immunity, Biological rhythms
Serotonergic synapse: PRKCA	-1,62E-01	7,78E-05	2,75E-03	Apoptosis, Angiogenesis, Cell adhesion, Adaptive immunity, Transcription regulation, Transcription, Immunity, Biological rhythms
Insulin secretion: PRKCA	-1,95E-01	8,64E-05	2,79E-03	Angiogenesis, Apoptosis, Cell adhesion, Adaptive immunity, Immunity, Transcription regulation, Transcription, Biological rhythms
Circadian entrainment: PRKCA	-1,71E-01	9,62E-05	2,86E-03	Angiogenesis, Apoptosis, Cell adhesion, Adaptive immunity, Transcription regulation, Transcription, Immunity, Biological rhythms
Ras signaling pathway: PRKCA	-2,20E-01	3,59E-04	6,69E-03	Apoptosis, Angiogenesis, Cell adhesion, Adaptive immunity, Transcription regulation, Transcription, Immunity, Biological rhythms
Signaling pathways regulating pluripotency of stem cells: CTNNB1*	4,53E-02	4,08E-04	6,69E-03	Transcription regulation, Transcription, Neurogenesis, Wnt signaling pathway, Cell adhesion
ErbB signaling pathway: PRKCA	-2,36E-01	4,91E-04	7,82E-03	Apoptosis, Angiogenesis, Cell adhesion, Adaptive immunity, Transcription regulation, Transcription, Immunity, Biological rhythms
cAMP signaling pathway: AFDN	3,36E-01	1,55E-03	2,03E-02	Cell adhesion

Subpathway_name	log2(FC)	Pvalue	FDR	UniprotKB_functions
Thyroid hormone signaling pathway: ATP1B4	-8,51E-02	2,08E-03	2,51E-02	Ion transport, Transcription regulation, Transcription, Transport, Sodium/potassium transport, Sodium transport, Potassium transport, Cell adhesion
Leukocyte transendothelial migration: BCAR1	-1,34E-01	3,54E-03	4,01E-02	Cell adhesion
Ras signaling pathway: PIK3R5*	-9,98E-02	4,52E-03	4,86E-02	Angiogenesis, Endocytosis, Autophagy, Cell adhesion, Chemotaxis, Adaptive immunity, Inflammatory response, Innate immunity, Differentiation, Immunity, Stress response, Host-virus interaction, Protein transport, Transport
Progesterone-mediated oocyte maturation: PIK3R5	-9,28E-02	4,63E-03	4,87E-02	Angiogenesis, Endocytosis, Autophagy, Cell adhesion, Chemotaxis, Inflammatory response, Adaptive immunity, Innate immunity, Differentiation, Immunity, Stress response, Protein transport, Host-virus interaction, Transport

Table S5. HiPathia analysis on RM82-shENG with shared cell adhesion UniprotKB function.

Antibody	Manufacturer	Reference
FAK1	CST	3285
FAK1 P Y397	CST	3283
CamKII P Thr286	CST	3361
MEK1/2	CST	9122
MEK1/2 P Ser217/221	CST	9154
PI3 Kinase p110-alpha	CST	4249
PKC (pan) P Ser660 (beta-2)	CST	9371
PKC-zeta	CST	9372
PKC-zeta/lambda P Thr410/403	CST	9378
ErbB-1/EGFR	CST	2232
EGFR P Tyr1173	CST	4407
Akt P Thr308	CST	2965
Src	CST	2109
Akt	CST	9272
Akt P Ser473	CST	4060
beta-actin	CST	4970
E-Cadherin	CST	3195
beta-Catenin	CST	9562
beta-Catenin P Ser33,Ser37,Thr41	CST	9561
beta-Catenin P Thr41,Ser45	CST	9565
VEGFR P Tyr1175	CST	2478
PTEN	CST	9552
PTEN P Ser380,Thr382,Thr383	CST	9554
GSK-3-alpha/beta P Ser21/Ser9	CST	9331
mTOR P Ser2448	CST	2971
PLC-gamma1 P Tyr783	CST	2821
Src (family) P Tyr416	CST	2101
Smad2/3 P Ser465/Ser423,Ser467/Ser425	CST	8828
Smad1/5 P Ser463/Ser465	CST	9516
EGFR P Tyr1086	Thermo Fisher Scientific	36-9700
GSK-3-beta P Ser9	CST	9336
GSK-3-beta	CST	9315
PKC-alpha	Beckton Dickinson	610108
PLC-gamma1	CST	2822
VEGFR P Tyr951	CST	4991
VEGFR P Tyr1059	CST	3817
Calmodulin	Calbiochem	NB12
PDGFR P Tyr1021	CST	2227
PDGFR P Tyr751	CST	4549
Zyxin	CST	3553

Antibody	Manufacturer	Reference
ILK1 (4G9)	CST	3856
Akt P Ser473	CST	9271
Akt P Thr308	CST	9275
EGFR P Y992	CST	2235
EGFR P Y1086	CST	2234
mTOR (7C10)	CST	2983
Integrin Beta 1 [EP1041Y]	Abcam	ab52971
mTOR P S2448 [EP2426(2)Y]	Abcam	ab109268
Smad2 (C86F7)	CST	3122
Caveolin-1 (D46G3) XP R	CST	3267
Integrin alpha 4	CST	4600
Integrin beta3	CST	4702
Integrin beta4	CST	4707
Smad1 (D59D7)	CST	6944
EphA2 (D4A2)	CST	6997
beta-Tubulin	Abcam	ab6046
PKC-alpha P Thr638	Abcam	ab32502
GAPDH	Abcam	ab9484
PKC-gamma P Thr514	GeneTex	GTX25778
mTOR P Ser2481	Millipore (Upstate)	09-343SP

Table S6. Antibodies used in RPPA.

ID	Description	*sgMMP14*	NES	pvalue	p.adjust	qvalues
GO:0000226	microtubule cytoskeleton organization	RM82	-1,53	3,39E-03	1,33E-01	1,19E-01
		SKNMC	-1,42	1,31E-03	1,78E-01	1,65E-01
GO:0034333	adherens junction assembly	RM82	-1,43	2,70E-02	2,69E-01	2,41E-01
		SKNMC	-1,83	1,64E-03	1,78E-01	1,65E-01
GO:0051893	regulation of focal adhesion assembly	RM82	-1,50	2,21E-02	2,46E-01	2,20E-01
		SKNMC	-1,78	6,57E-03	2,46E-01	2,29E-01
GO:0033627	cell adhesion mediated by integrin	RM82	-1,49	2,43E-02	2,58E-01	2,32E-01
		SKNMC	-1,66	8,20E-03	2,46E-01	2,29E-01
GO:0031012	extracellular matrix	RM82	1,61	1,40E-03	7,16E-02	6,27E-02
		SKNMC	-2,11	1,29E-03	1,00E-01	8,92E-02
GO:0030545	receptor regulator activity	RM82	1,80	1,42E-03	1,26E-01	1,12E-01
		SKNMC	-1,54	2,63E-03	2,73E-01	2,61E-01
GO:0050840	extracellular matrix binding	RM82	1,83	1,69E-03	1,26E-01	1,12E-01
		SKNMC	-1,70	8,67E-03	2,88E-01	2,75E-01
GO:0070006	metalloamino peptidase activity	RM82	-1,87	6,96E-03	1,62E-01	1,45E-01
		SKNMC	-1,70	1,84E-02	3,66E-01	3,49E-01
GO:0005201	extracellular matrix structural constituent	RM82	1,49	9,58E-03	1,75E-01	1,56E-01
		SKNMC	-2,13	1,52E-03	2,73E-01	2,61E-01
WP3888	VEGFA-VEGFR2 Signaling	RM82	-1,35	1,45E-02	1,83E-01	1,64E-01
		SKNMC	-1,41	5,19E-03	1,80E-01	1,68E-01

Table S7. GSEA from RM82- and SKNMC-sgMMP14 models

Gene name	Protein name	ENG / IgG	
		LFQ Ratio	p-value
**ACTR1A**	Alpha-centractin	1,00E+08	**3,66E-03**
**ILKAP**	Integrin-linked kinase-associated serine/threonine phosphatase 2C	3,70E+07	**7,66E-06**
**APOF**	Apolipoprotein F	1,50E+07	**2,44E-02**
**DCTN3**	Dynactin subunit 3	1,20E+07	**1,98E-02**
**DCTN4**	Dynactin subunit 4	8,50E+06	**2,21E-03**
**PGAM5**	Serine/threonine-protein phosphatase PGAM5, mitochondrial	7,90E+06	**4,93E-03**
**PPIL1**	Peptidyl-prolyl cis-trans isomerase-like 1	7,50E+06	**3,45E-02**
**ADORA3**	Adenosine receptor A3	3,00E+06	**2,67E-02**
**sep-09**	Septin-9	2,70E+06	**2,20E-04**
**VRK1**	Serine/threonine-protein kinase VRK1	9,60E+05	**3,27E-02**
**CEP290**	Centrosomal protein of 290 kDa	9,30E+03	**2,28E-02**
**ENG**	**Endoglin**	**3,20E+02**	**6,13E-03**
**IQCB1**	IQ calmodulin-binding motif-containing protein 1	8,80E+01	**2,20E-02**
**SORBS2**	Sorbin and SH3 domain-containing protein 2	4,20E+01	**2,64E-05**
**CALM3**	Calmodulin-3	3,00E+01	**7,23E-03**
**COL1A1**	Collagen alpha-1(I) chain	2,10E+01	**2,78E-02**
**CRABP1**	Cellular retinoic acid-binding protein 1	1,90E+01	**1,22E-04**
**DCTN2**	Dynactin subunit 2	1,70E+01	**1,57E-03**
**IGKV3OR2-268**	Ig kappa chain V-III region POM	8,60E+00	**7,41E-05**
**IARS**	Isoleucine--tRNA ligase, cytoplasmic	5,30E+00	**1,40E-02**
**PLAA**	Phospholipase A-2-activating protein	5,20E+00	**1,98E-02**

Table S8. Table depicting the most enriched ENG -interactors identified by IP/MS. LFQ ratios ENG/IgG and associated *p*-values are shown. Herein, three biological replicates were used for each individual experiment. Statistical analysis was performed by paired two-tailed Student's *t*- test.
