# Supplementary figures and images for: Endoglin and MMP14 Contribute to Ewing Sarcoma Spreading by Modulation of Cell–Matrix Interactions
Source: Int J Mol Sci. 2022 Aug 4;23(15):8657. doi: 10.3390/ijms23158657 (PMC9369355; doi:10.3390/ijms23158657)

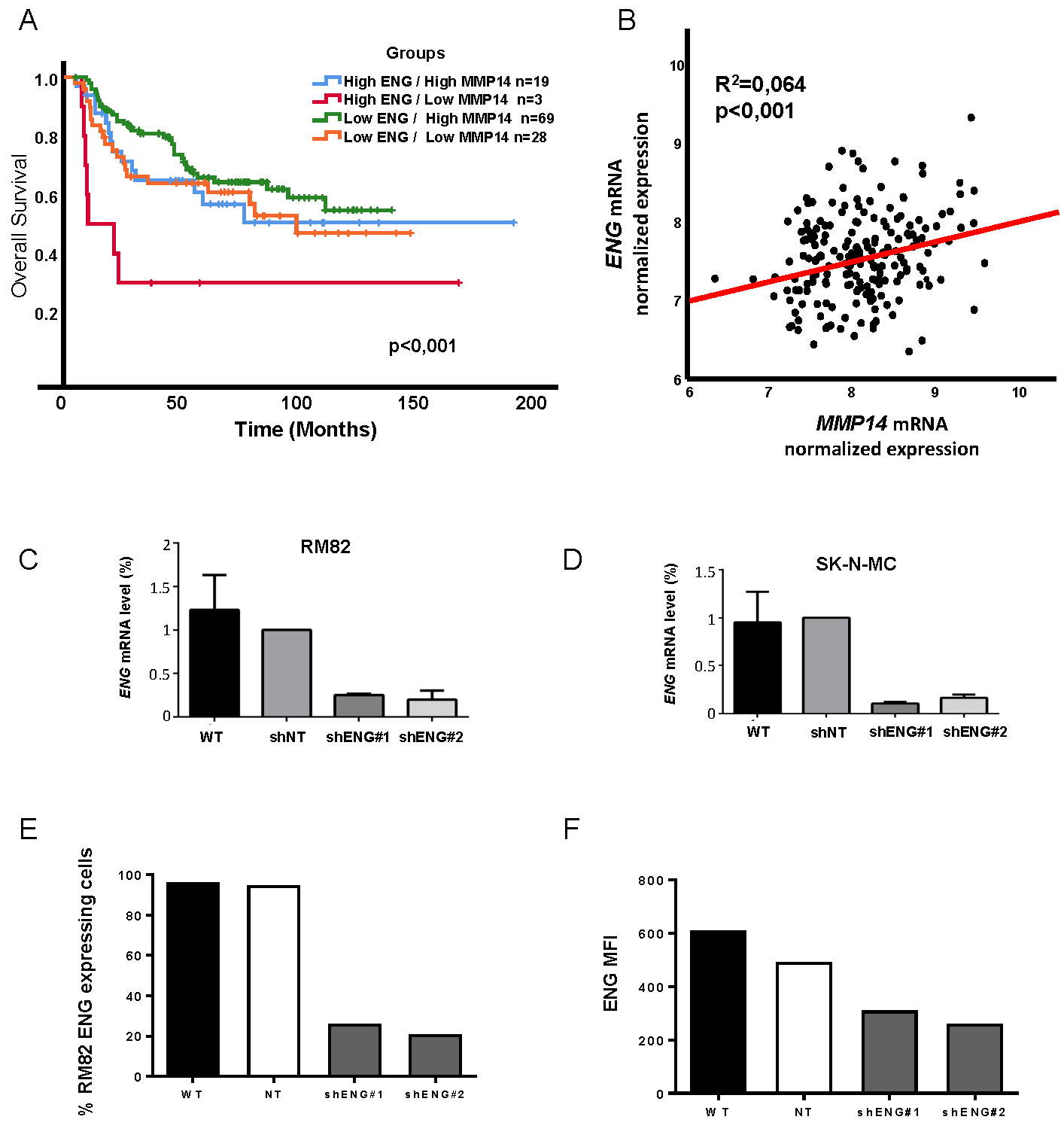

Supplement: Supplementary file 1 [file ijms-23-08657-s001.zip › Figure S1.png]

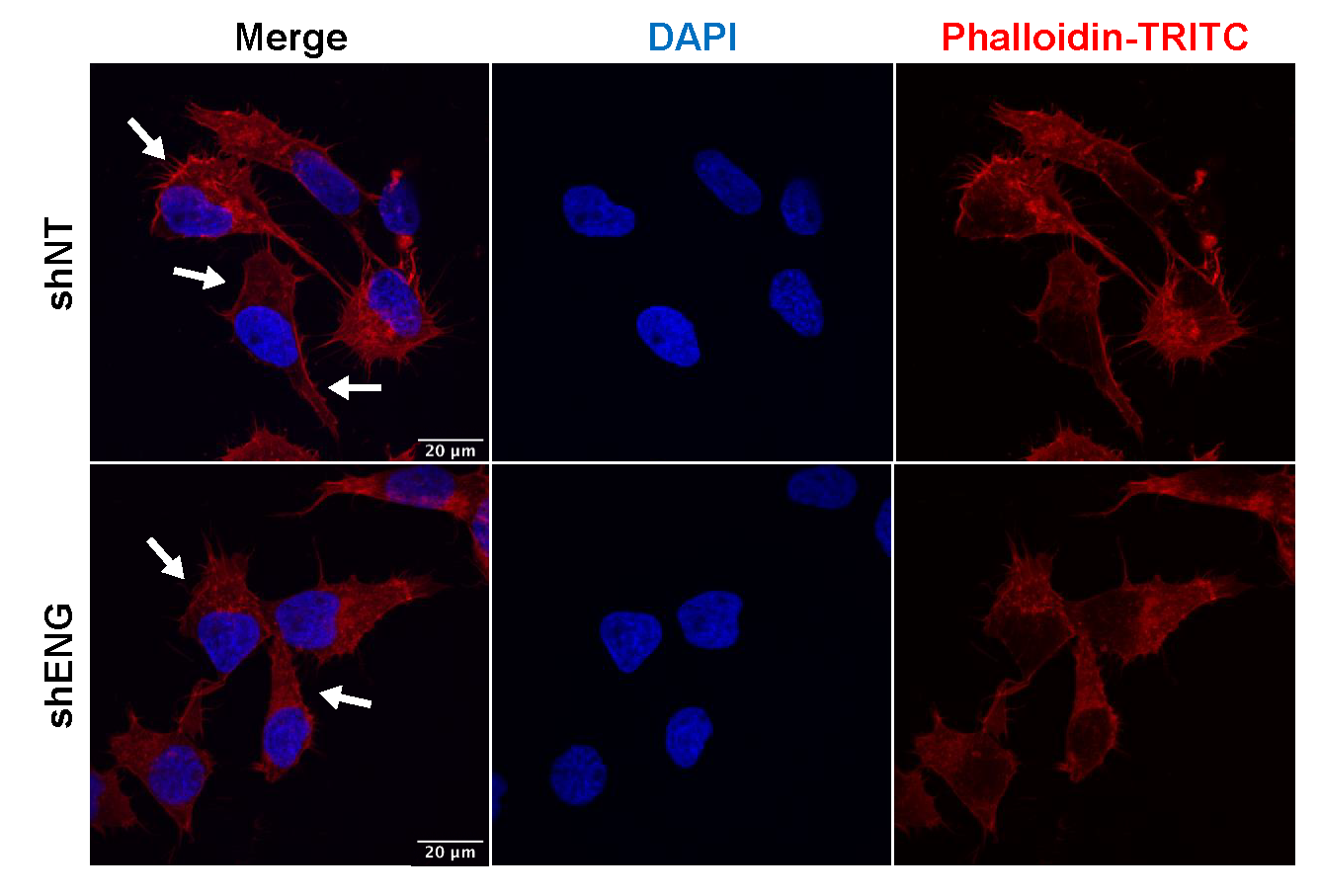

Supplement: Supplementary file 1 [file ijms-23-08657-s001.zip › Figure S10.png]

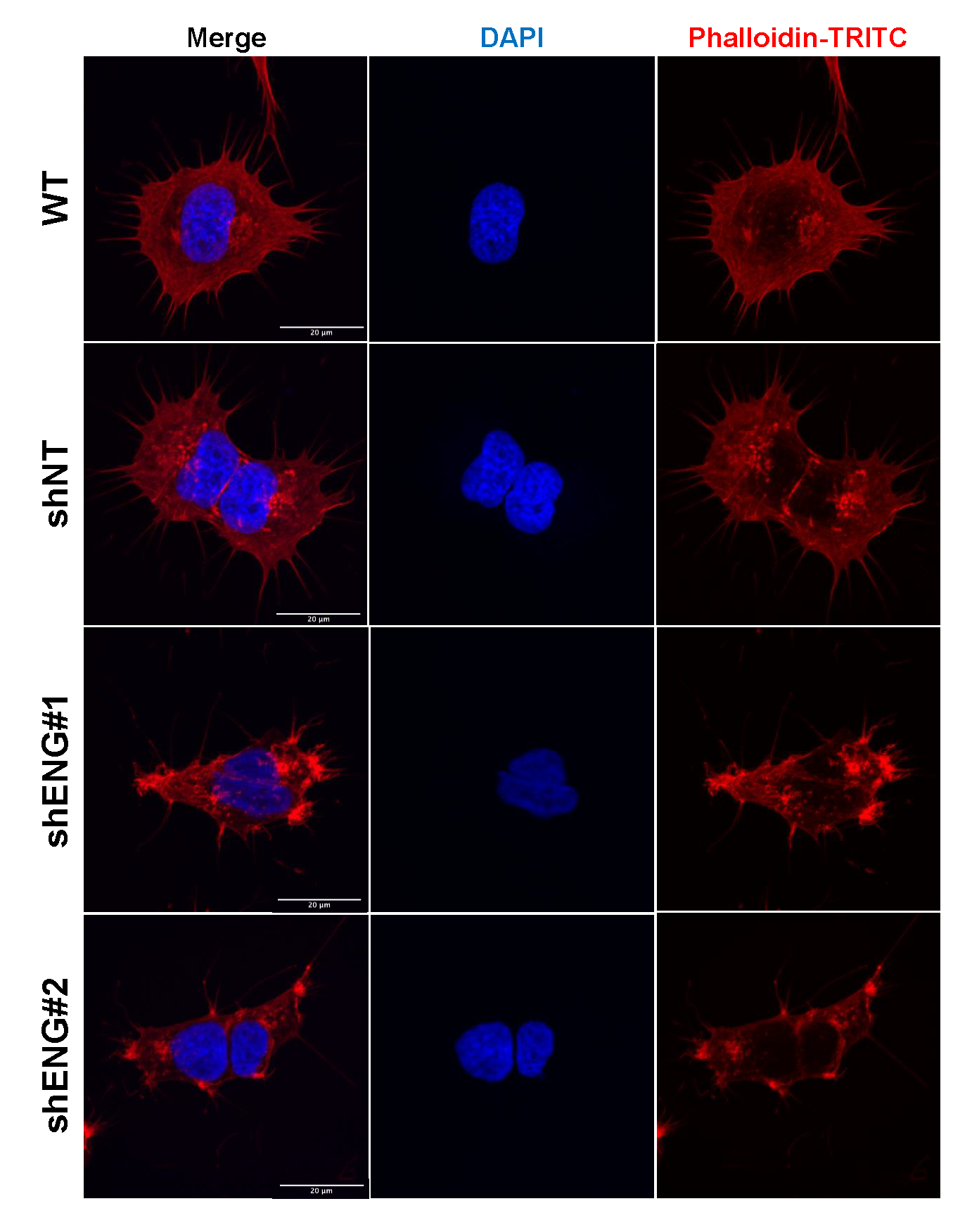

Supplement: Supplementary file 1 [file ijms-23-08657-s001.zip › Figure S11.png]

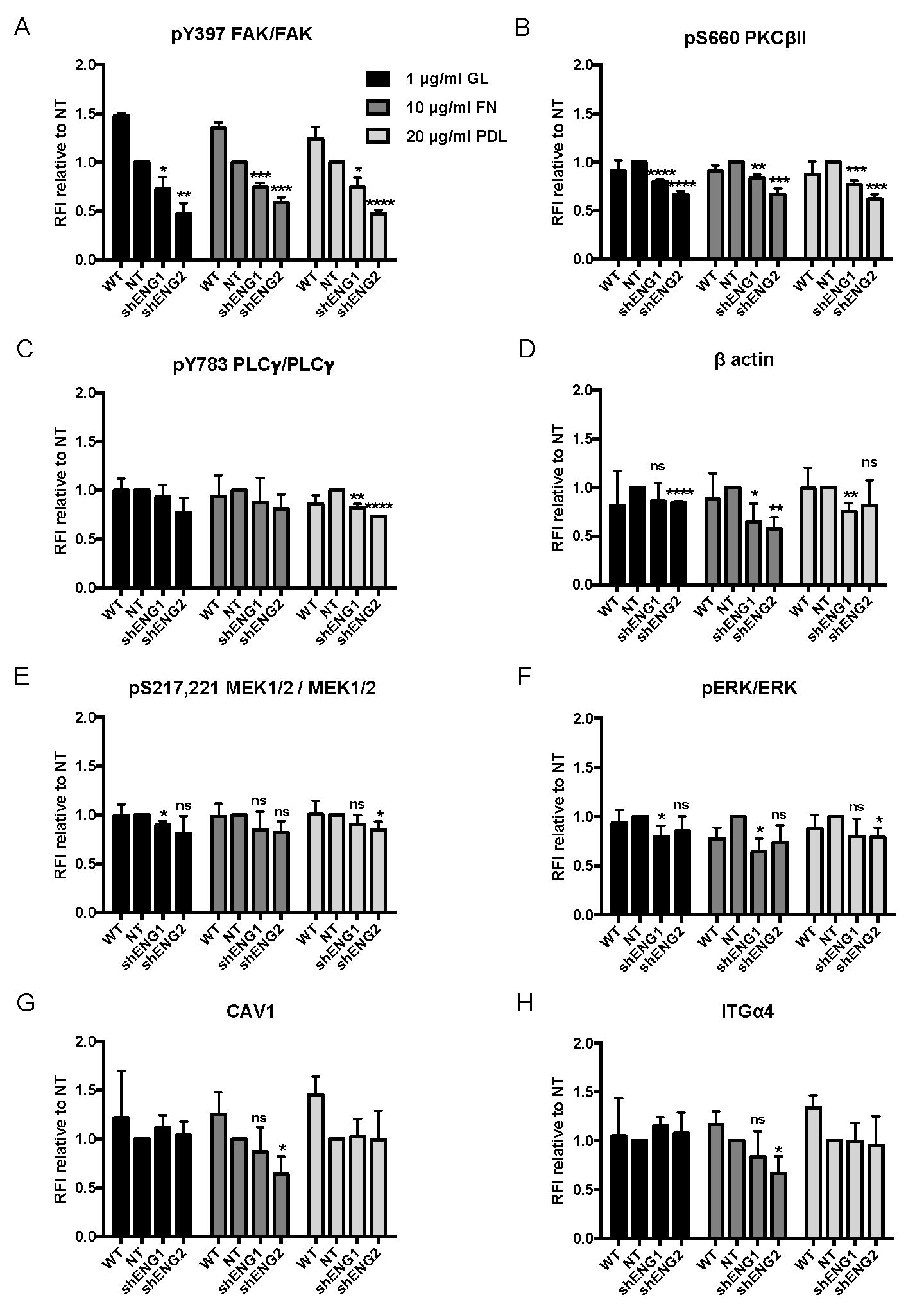

Supplement: Supplementary file 1 [file ijms-23-08657-s001.zip › Figure S12.png]

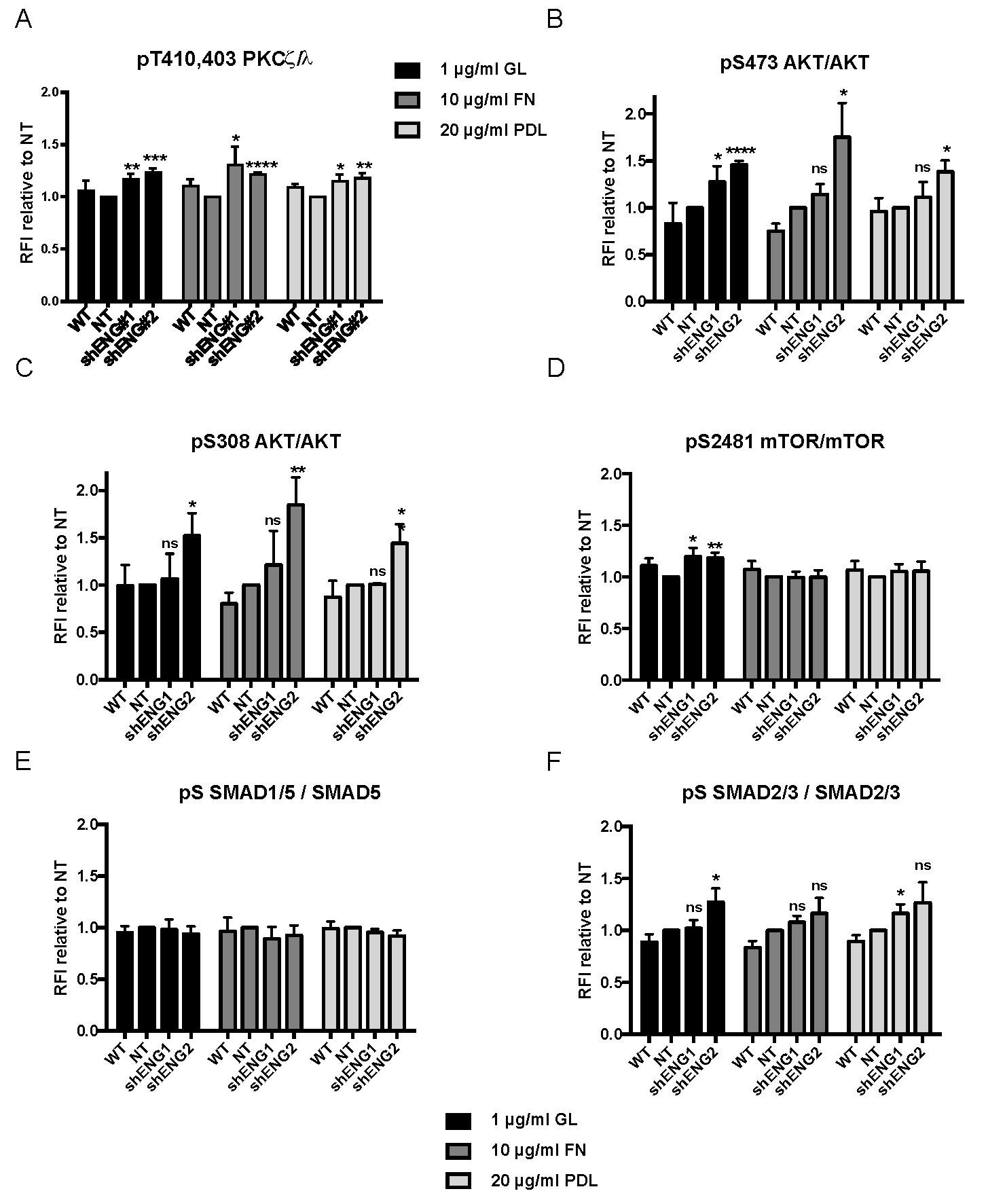

Supplement: Supplementary file 1 [file ijms-23-08657-s001.zip › Figure S13.png]

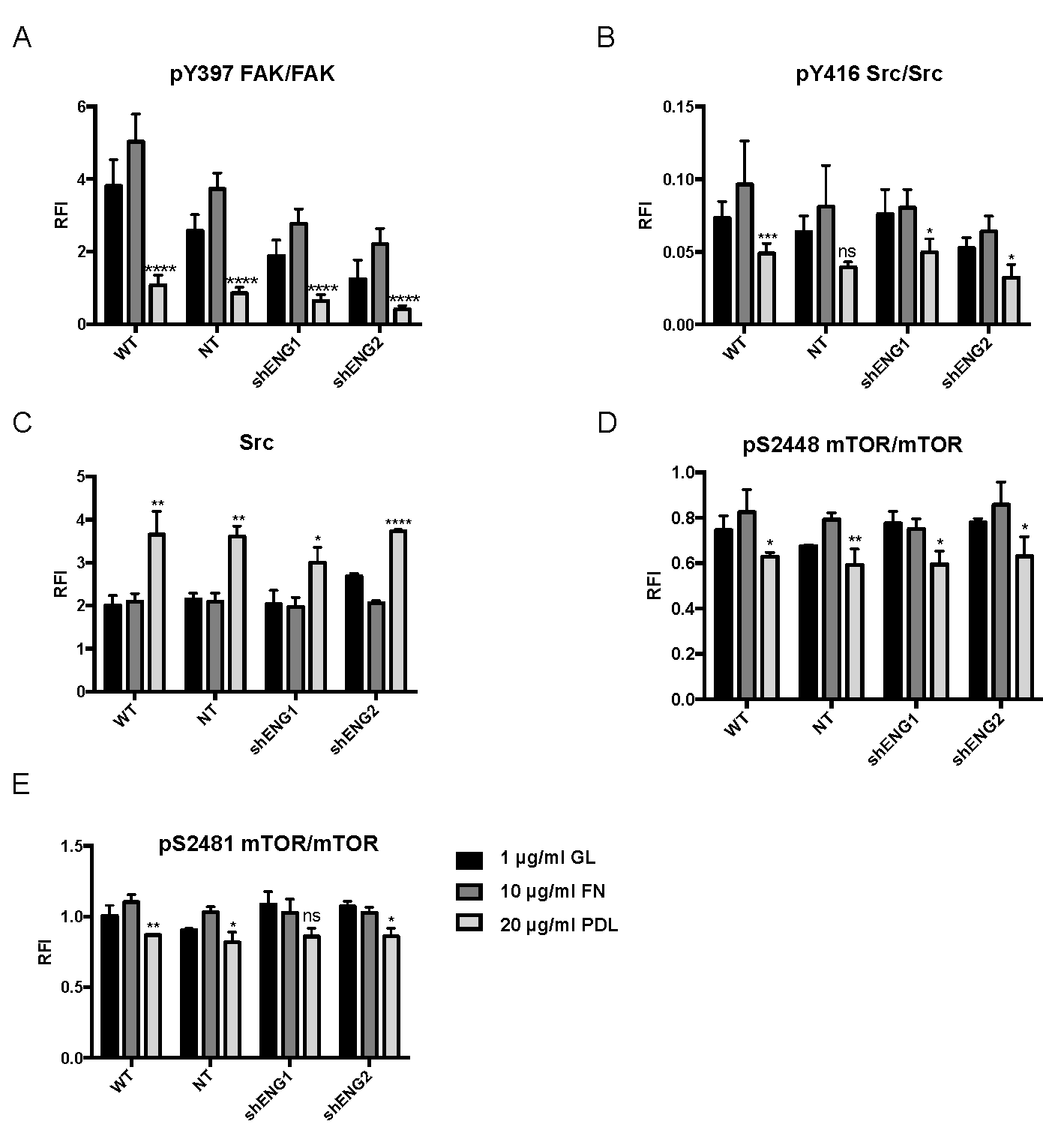

Supplement: Supplementary file 1 [file ijms-23-08657-s001.zip › Figure S14.png]

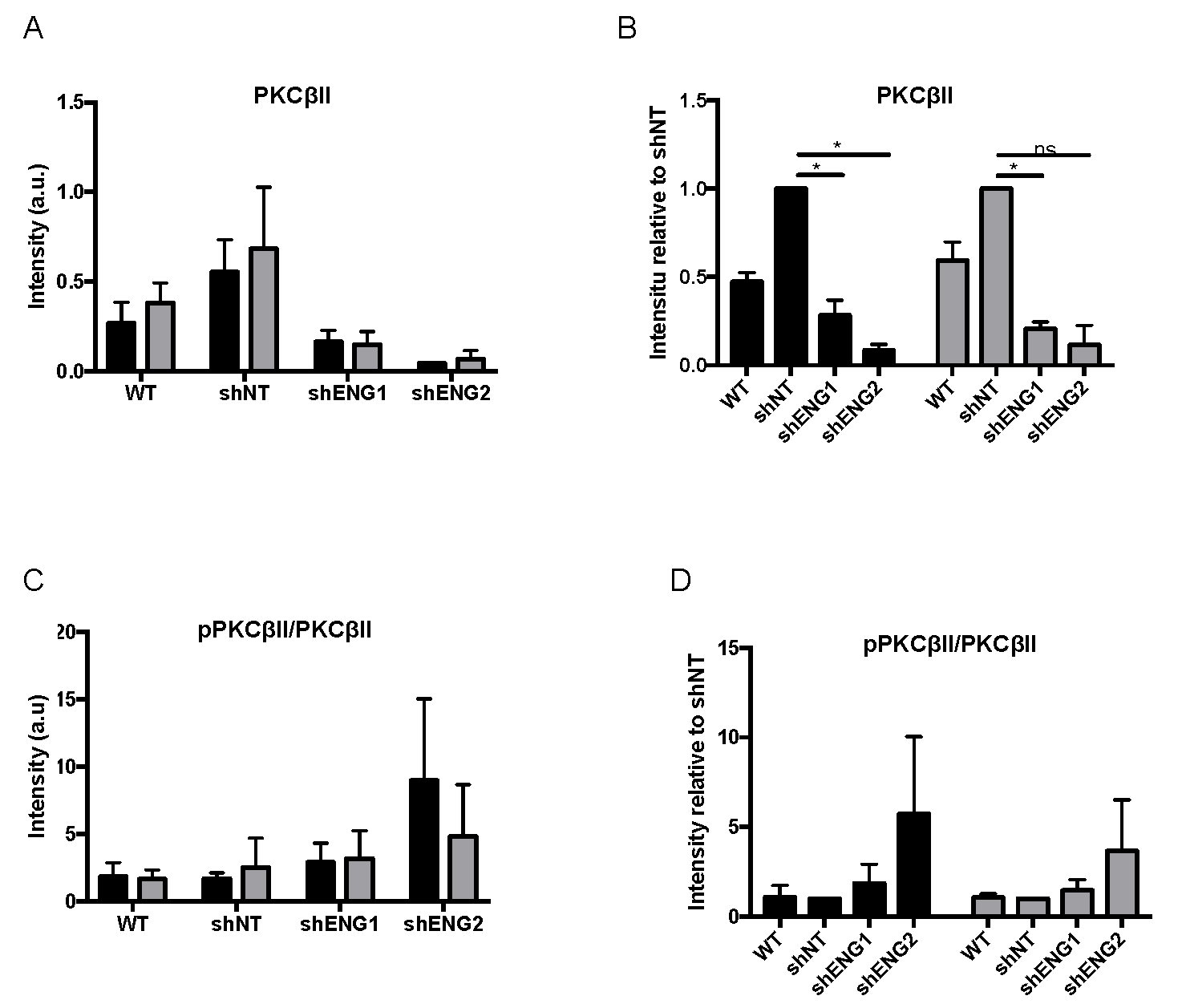

Supplement: Supplementary file 1 [file ijms-23-08657-s001.zip › Figure S15.png]

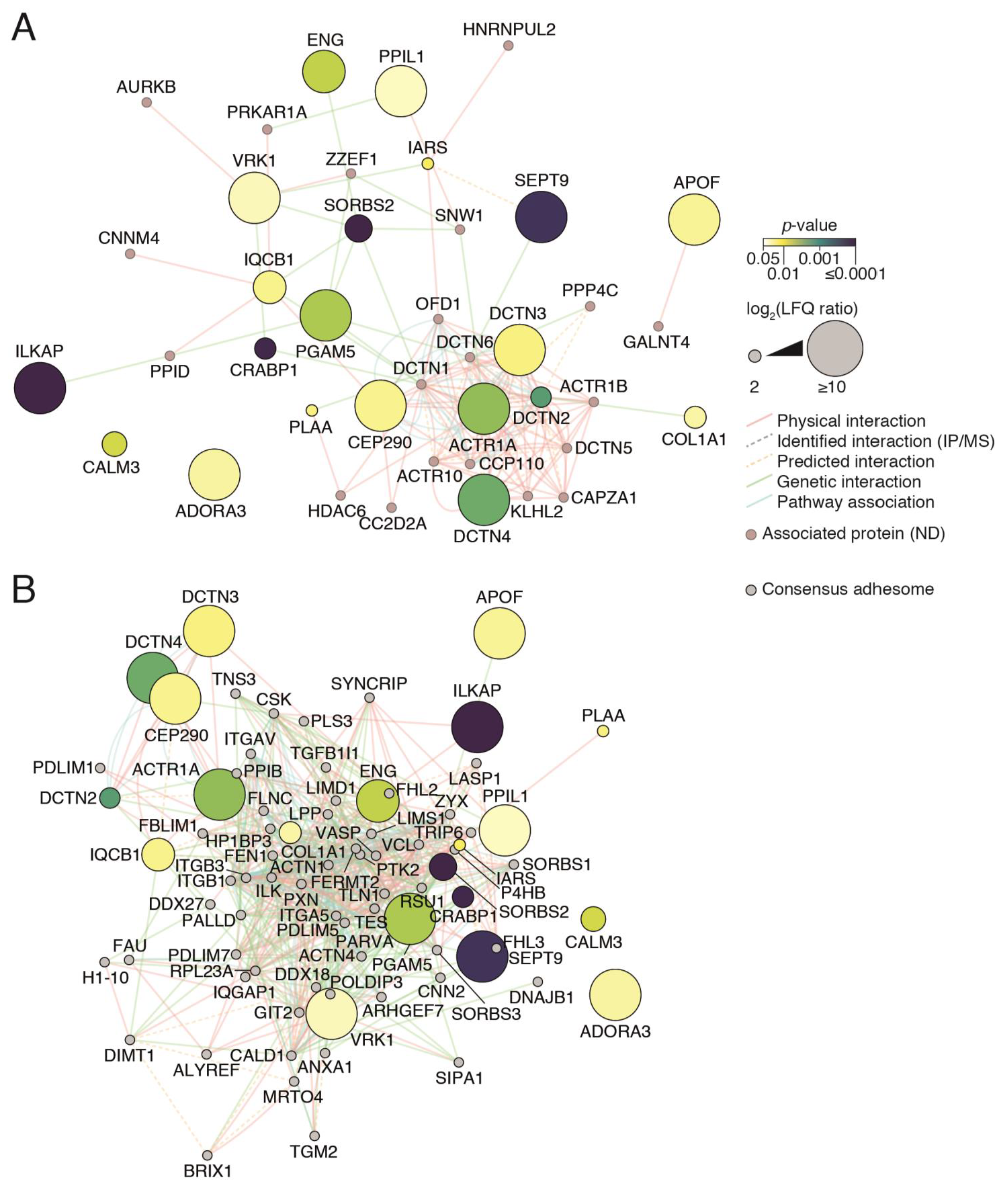

Supplement: Supplementary file 1 [file ijms-23-08657-s001.zip › Figure S16.png]

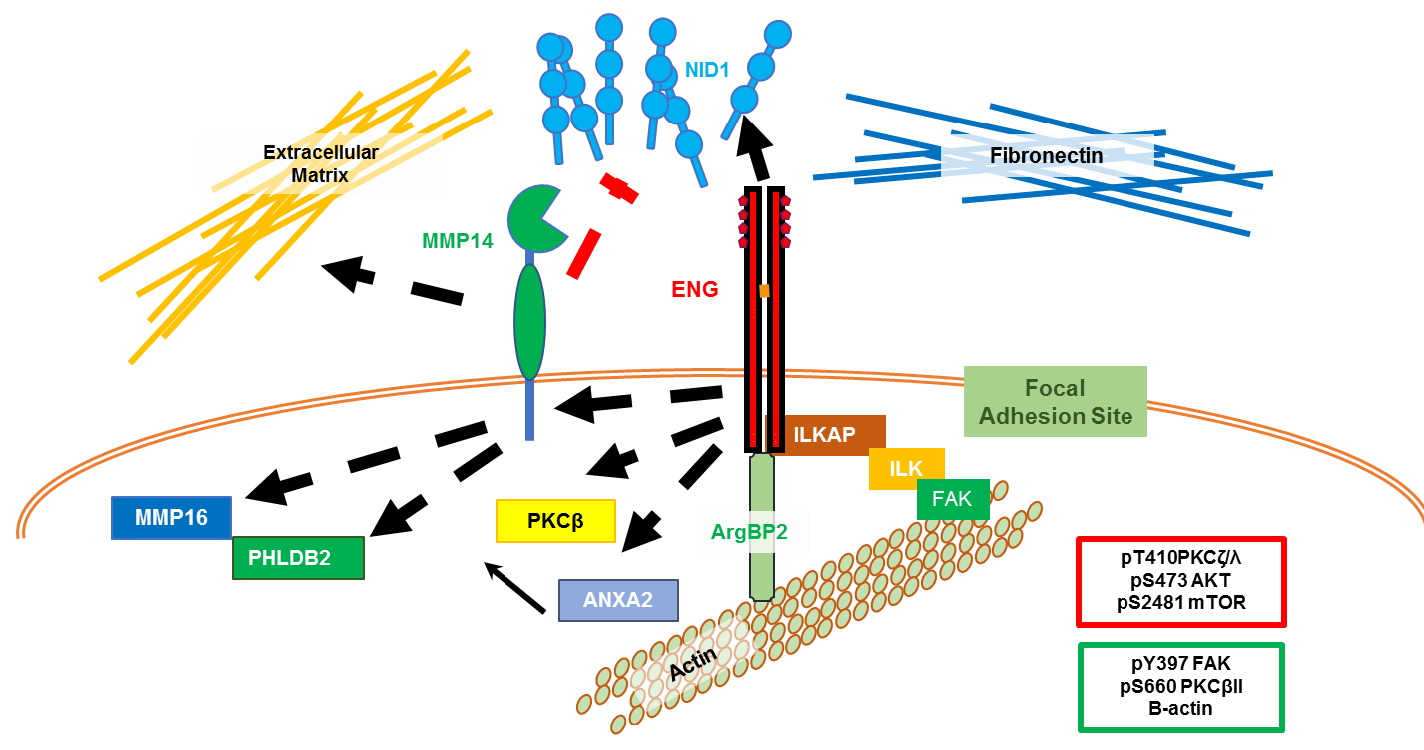

Supplement: Supplementary file 1 [file ijms-23-08657-s001.zip › Figure S17.png]

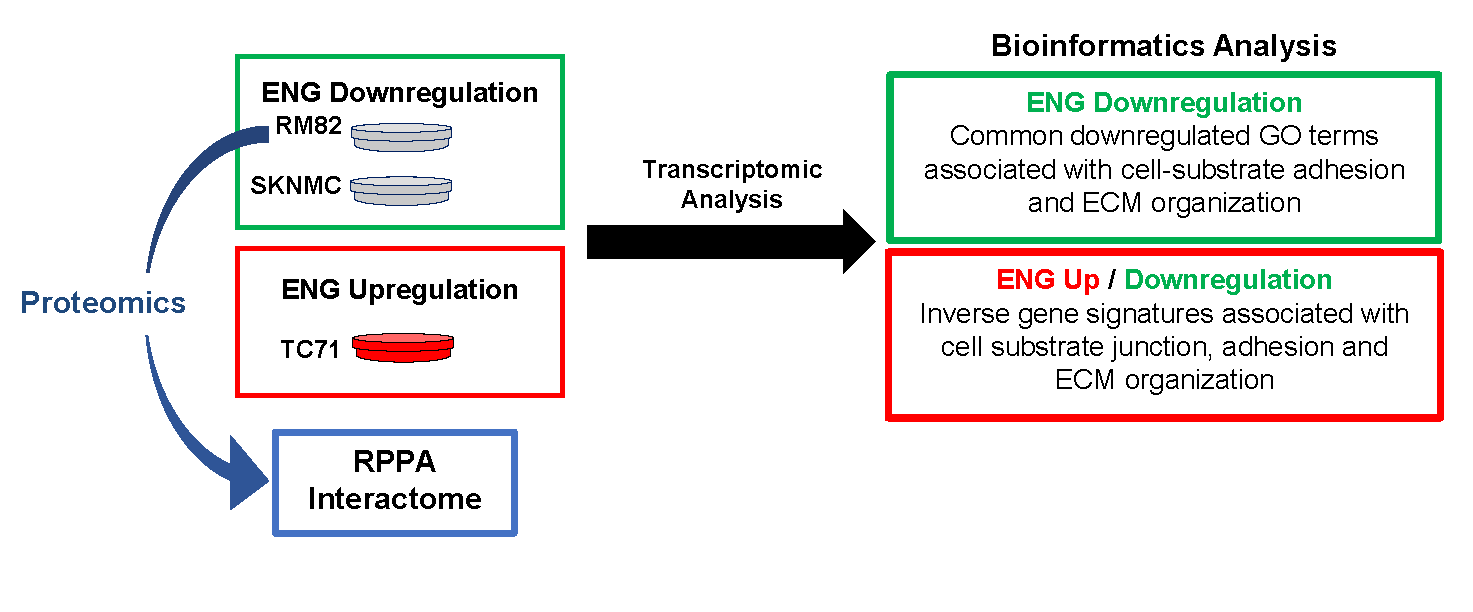

Supplement: Supplementary file 1 [file ijms-23-08657-s001.zip › Figure S2.png]

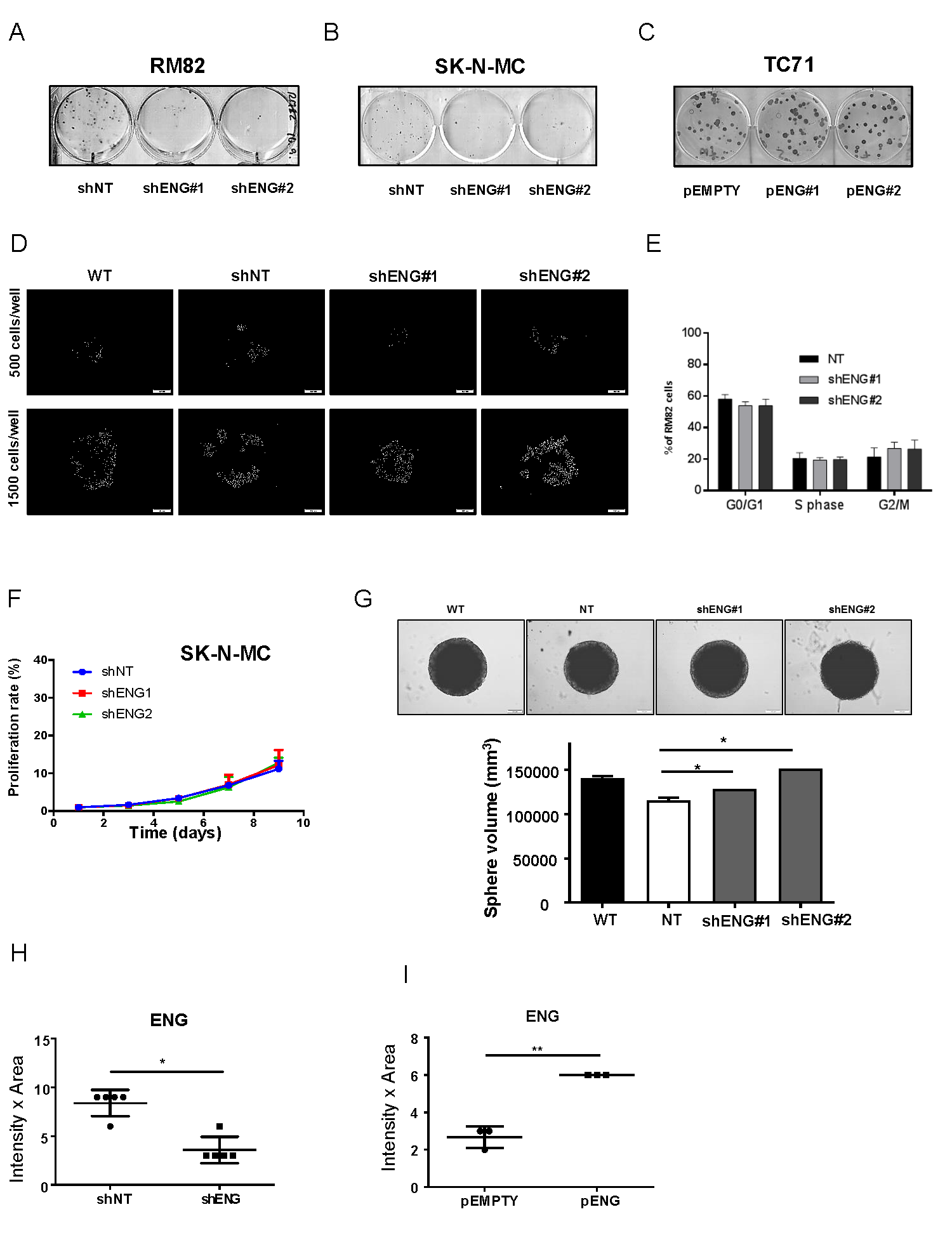

Supplement: Supplementary file 1 [file ijms-23-08657-s001.zip › Figure S3.png]

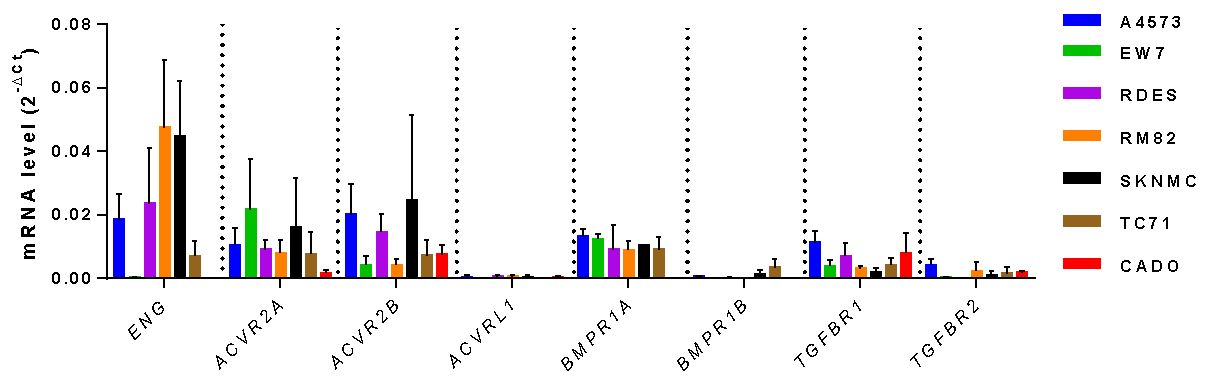

Supplement: Supplementary file 1 [file ijms-23-08657-s001.zip › Figure S4.png]

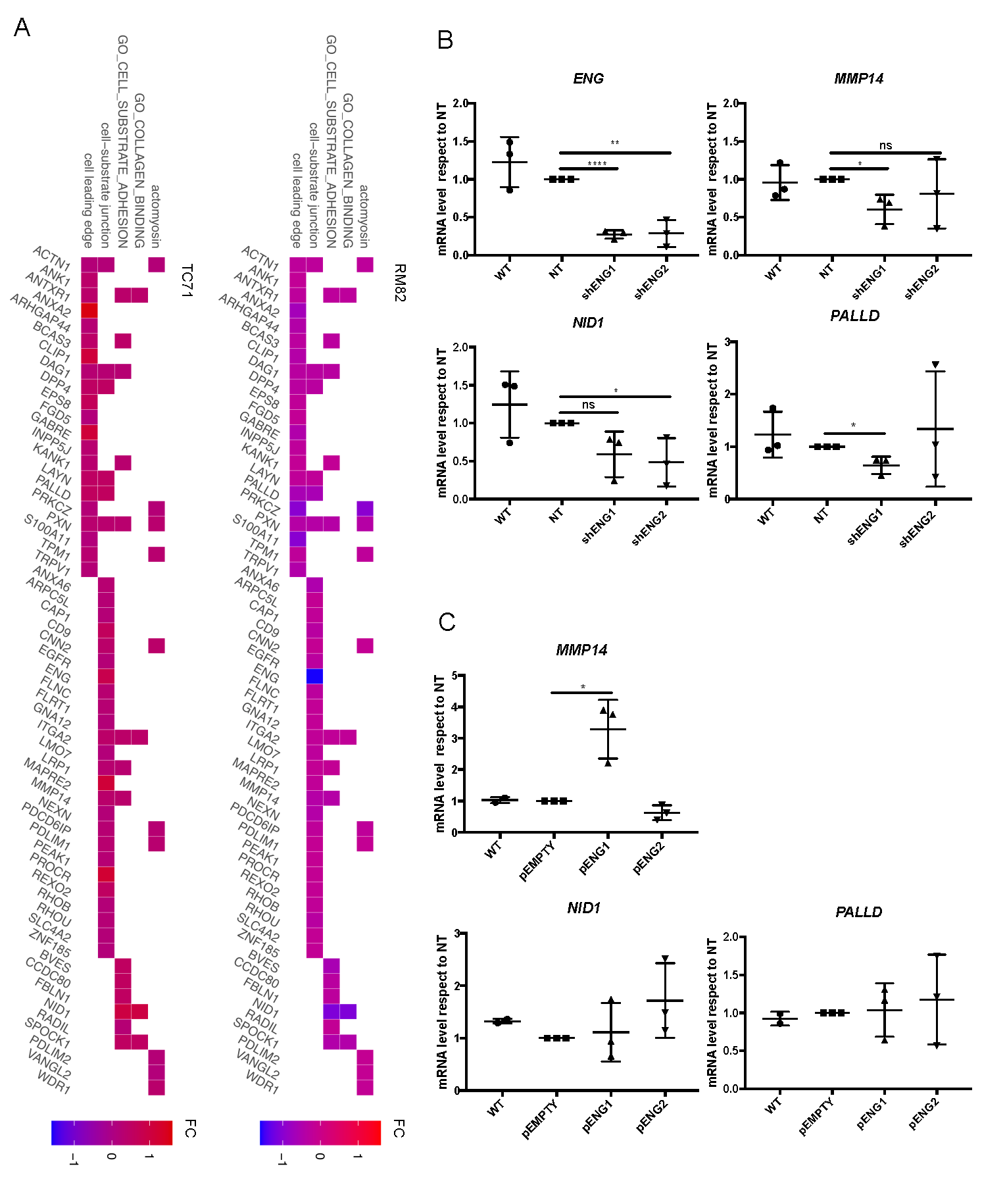

Supplement: Supplementary file 1 [file ijms-23-08657-s001.zip › Figure S5.png]

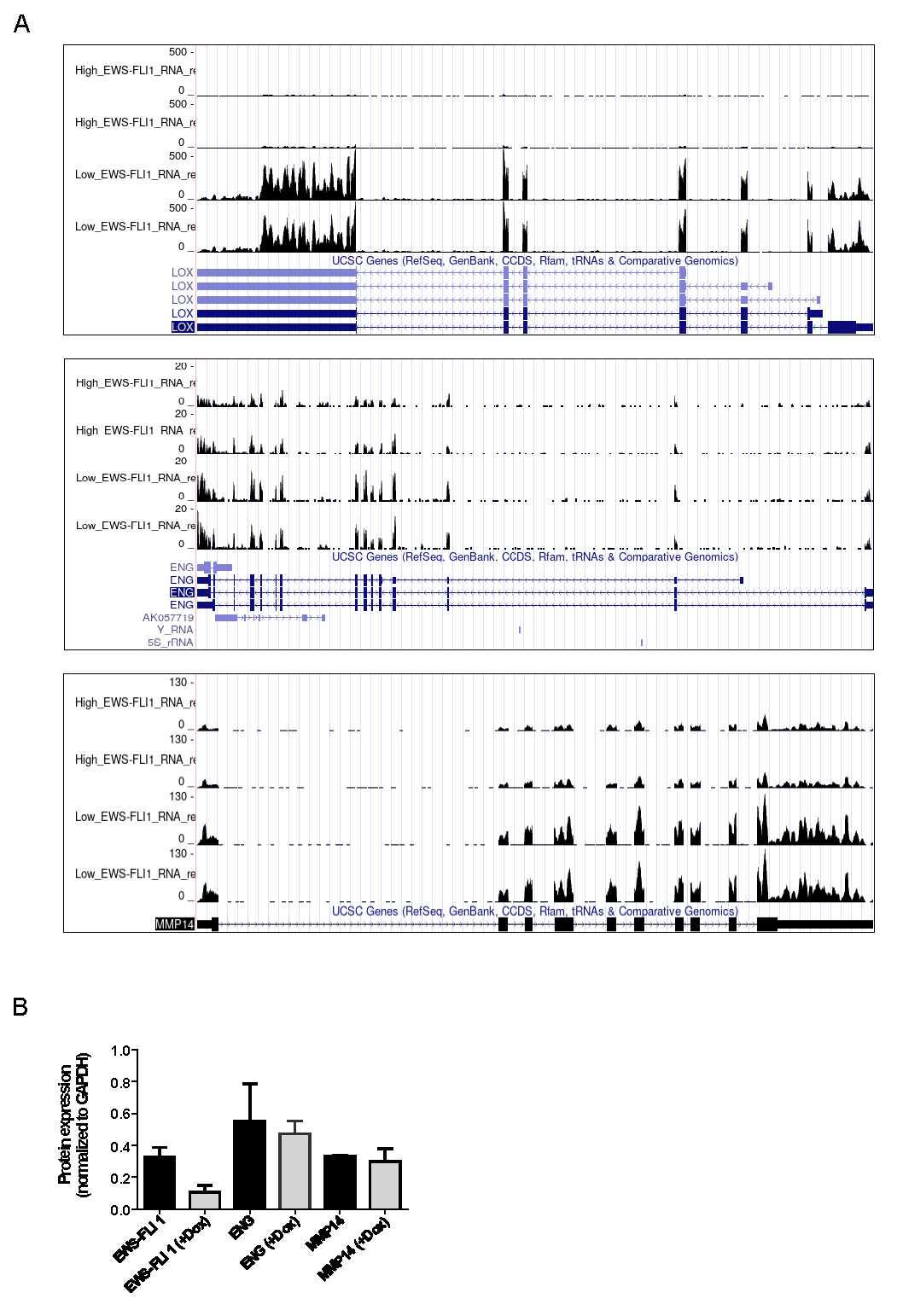

Supplement: Supplementary file 1 [file ijms-23-08657-s001.zip › Figure S6.png]

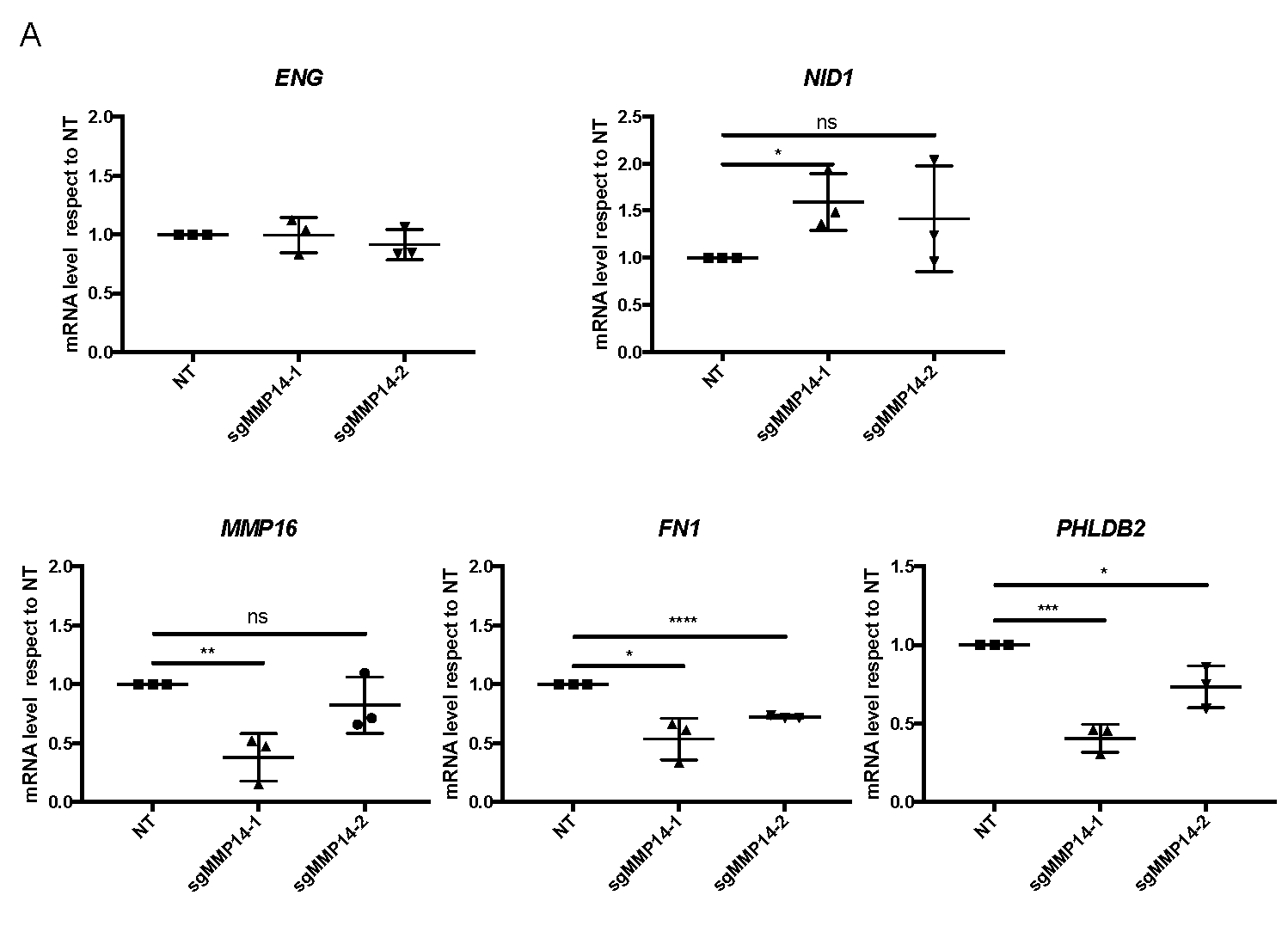

Supplement: Supplementary file 1 [file ijms-23-08657-s001.zip › Figure S7.png]

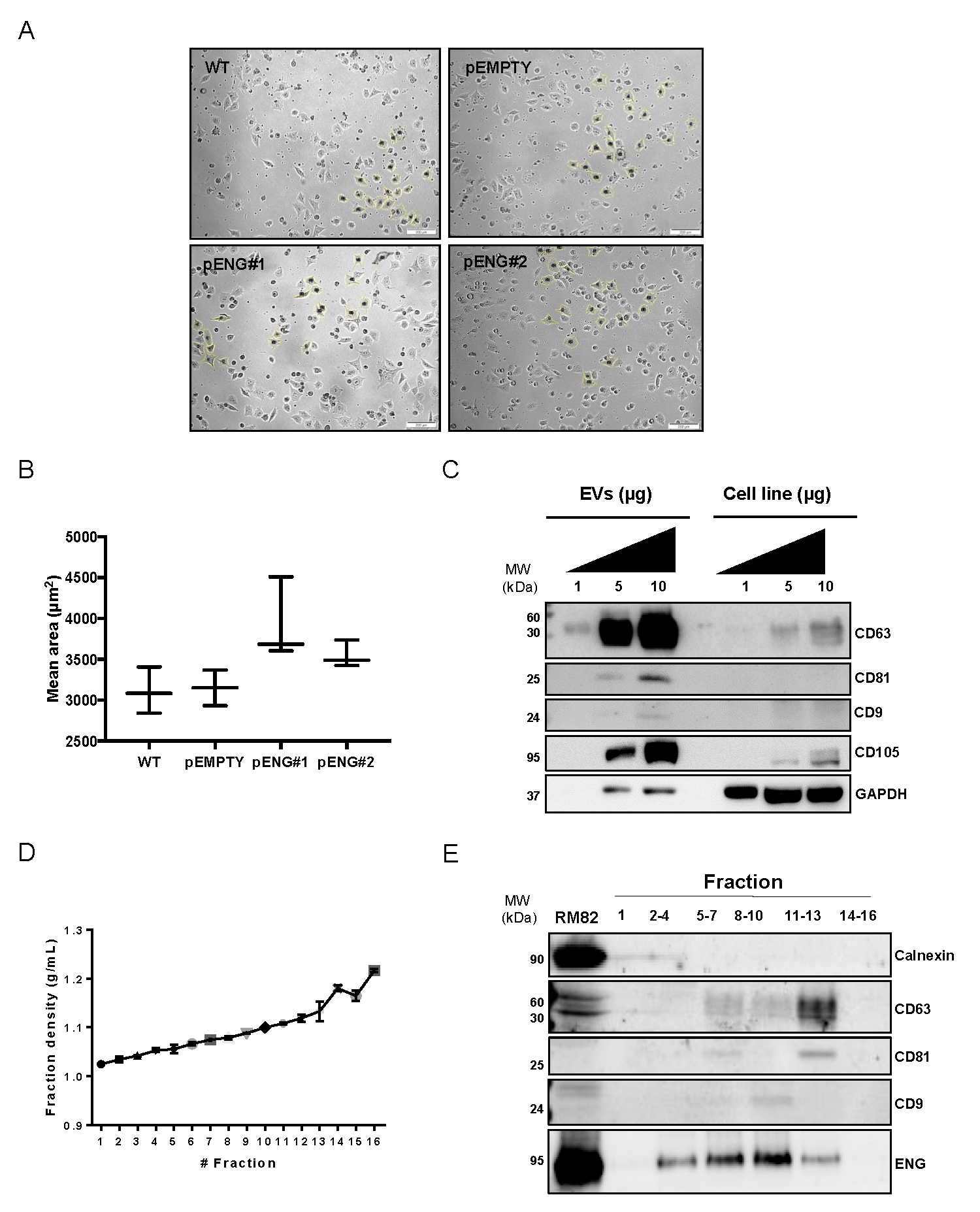

Supplement: Supplementary file 1 [file ijms-23-08657-s001.zip › Figure S8.png]

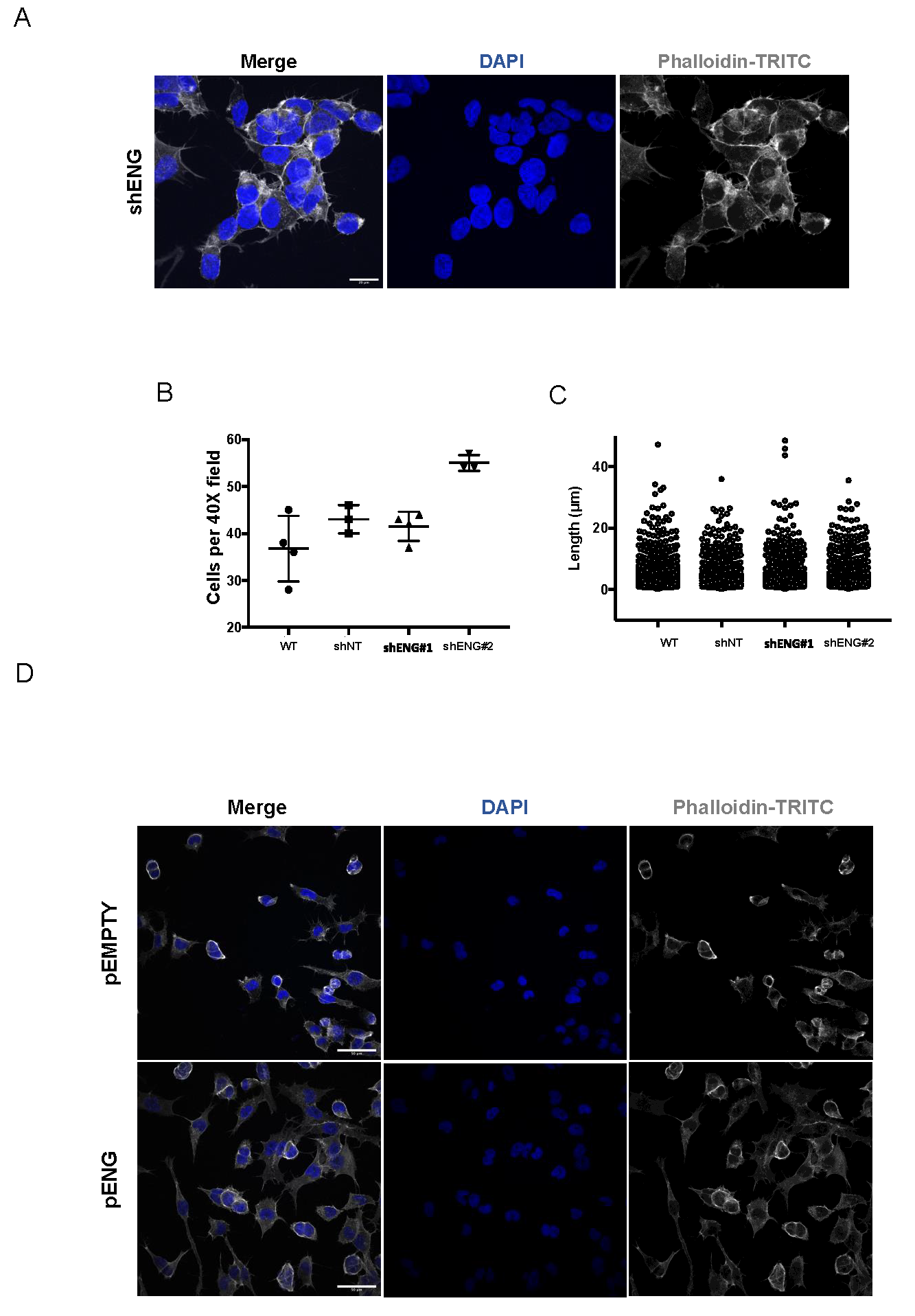

Supplement: Supplementary file 1 [file ijms-23-08657-s001.zip › Figure S9.png]
